# Supplementary material for: GWAS for primary angle-closure glaucoma identifies loci related to ocular biometry and morphology
Source: Nat Commun. 2025 Nov 14;16:10003. doi: 10.1038/s41467-025-64949-z (PMC12618631; doi:10.1038/s41467-025-64949-z)
Supplement: Supplementary file 1 — Supplementary Information [file 41467_2025_64949_MOESM1_ESM.pdf]

nature communications

Supplementary information

GWAS for primary angle-closure glaucoma identifies loci related to ocular biometry and morphology

Robert N Luben, Mahantesh I Biradar, Kelsey V Stuart, Ruiqi Hu, Zihan Sun, Zheng Li, Ningli Wang, Tan Do, Chi Pui Pang, Masakazu Nakano, Kei Tashiro, Yoko Ikeda, Yuichi Tokuda, Masami Tanaka, Natsue Omi, Morio Ueno, Chie Sotozono, Shigeru Kinoshita, Kazuhiko Mori, Naris Kitnarong, Antonio Fea, Mônica B Melo, José Paulo C Vasconcellos, Vital P Costa, Monisha E Nongpiur, Ching Lin Ho, Shamira A Perera, Jamie E Craig, Antonia Kolovos, Ahmad Tajudin Liza-Sharmini, Edgar U Leuenberger, Ki Ho Park, Lingam Vijaya, Ronnie George, Tin Aung, C C Khor, Paul J Foster, Pirro Hysi, Anthony P Khawaja

Contents

Supplementary tables

Supplementary Table 1: Genome-wide significant lead variants at loci associated with PACG in UK Biobank European ancestry discovery GWAS and their association with PACG in combined European ancestry replication cohorts

Supplementary Table 2: Association of variants identified in conditional analysis from PACG discovery GWAS for each replication cohort

Supplementary Table 3: Lead variants from the multi-ancestry meta-analysis of 21 European and Asian cohorts (9217 PACG cases, 788,285 controls); comparison with European and Asian results

Supplementary Table 4: Lead variants from the multi-ancestry meta-analysis of 21 European and Asian cohorts (9217 PACG cases, 788,285 controls); reported associations with refraction or iris morphology

Supplementary Table 5: Genetic correlation analyses with traits related to anthropometry, education, glaucoma, pigmentation and refraction, in Europeans

Supplementary Table 6: Two-sample Mendelian randomisation for axial length, eye colour and refractive error on PACG

Supplementary Table 7: Logistic regression models of PACG and PAC cases and controls in EPIC-Norfolk

Supplementary Table 8: The association of PACG-related traits in EPIC-Norfolk with quintiles of polygenic risk score derived from the PACG European ancestry meta-analysis

Supplementary Table 9: Dominant and recessive models for GWAS significant loci from the European meta-analysis

Supplementary figures

Supplementary Figure 1: Forest plot presenting the associations with PACG for lead discovery GWAS variants in the discovery and replication cohorts

Supplementary Figure 2: Regional association analysis for PACG genome-wide significant loci from PACG European meta-analysis

Supplementary Figure 3: Manhattan plot for GWAS of the European meta-analysis

Supplementary Figure 4: Comparison of the effect size of genome-wide significant loci in the European meta-analyses with loci in the Asian meta-analyses

Supplementary Figure 5: Multi-ancestry meta-analysis Manhattan plot of European and Asian cohorts

Supplementary Figure 6: Genetic correlations of PACG-related traits with PACG

Supplementary Figure 7: Results for Mendelian randomisation experiment examining the potential causal effect of refractive error on PACG

Supplementary Figure 8: Results for Mendelian randomisation experiment examining the potential causal effect of eye colour on PACG

Supplementary Figure 9: Two-sample Mendelian randomisation leave-one-out analysis of eye colour loci and PACG

Supplementary Figure 10: Polygenic risk score PRS-A from the MTAG analysis adjusted for spherical equivalent applied to 47 PACG and PAC cases and 6,623 controls in the EPIC-Norfolk study

Supplementary Figure 11: Plots of PACG-related traits in EPIC-Norfolk with quintiles of polygenic risk score PRS-B derived from the PACG European ancestry meta-analysis

Supplementary Figure 12: Polygenic risk score PRS-B from the European meta-analysis applied to 47 PACG and PAC cases and 6,623 controls in the EPIC-Norfolk study

Supplementary Figure 13: Polygenic risk score PRS-A from the MTAG analysis applied to 137 PACG and APAC cases and 245 controls from replication cohort participants with non-European ancestry

Supplementary Figure 14: Simulations of GWAS sample size by number of discovered variants, AUROC and variance explained using the PACG European meta-analysis

Supplementary Figure 15: Principal Component Analysis of replication cohorts for the determination of European ancestry

Supplementary Figure 16: Quantile-quantile plot and lambda statistics for discovery GWAS and European meta-analysis

Supplementary Figure 17: Manhattan plot for GWAS of the UK Biobank discovery

Supplementary notes

Supplementary Note 1: Inclusion Criteria and PACG definitions for replication cohorts from Italy, UK, USA, Australia and Brazil

Supplementary Note 1.1 Definition for Acute Primary angle-closure (APAC)

Supplementary Note 1.2 Definition for Primary angle-closure glaucoma (PACG)

Supplementary Note 1.3 Cohort and specific recruitment details

Supplementary Note 2: Inclusion Criteria and PACG definitions for FinnGen and the EPIC-Norfolk Eye Study

Supplementary references

Supplementary tables

Supplementary Table 1: Genome-wide significant lead variants at loci associated with PACG in UK Biobank European ancestry discovery GWAS and their association with PACG in combined European ancestry replication cohorts

Variant	Chromosome: Position	Nearest Gene	Reference: Effect allele	Discovery			Replication		
				Odds ratio per allele	P-value	Effect allele frequency	Odds ratio per allele	P-value	Effect allele frequency
rs184176302	4:30545538	*PCDH7*	C:CT	0.44	**1.06×10^{-8}**	0.99	0.86	0.34	0.98
rs17245595	7:83604975	*SEMA3A*	C:T	1.22	**3.02×10^{-8}**	0.56	1.11	**6.60×10^{-3}**	0.57
rs11984688	8:52647065	*PXDNL*	A:C	0.51	**1.11×10^{-9}**	0.98	0.56	**1.24×10^{-5}**	0.98
rs746970	9:4217822	*GLIS3*	G:A	1.24	**2.29×10^{-8}**	0.66	1.18	**4.03×10^{-5}**	0.70
rs12913832	15:28365618	*HERC2*	G:A	1.29	**5.62×10^{-10}**	0.21	1.34	**7.18×10^{-9}**	0.16

Discovery used 1564 cases and 439,185 controls of European ancestry from UK Biobank. The replication used 1619 cases and 334,031 controls from six national cohorts: Australia, Brazil, Italy, Finland, UK and USA. Further details in Figure 1, Study design. The variant rs184176302 (chromosome 4, position 30545538, deletion CT->C) was not available in replication data and a variant rs115261251 (r^2=0.45), was used in its place.

Supplementary Table 2: Association of variants identified in conditional analysis from PACG discovery GWAS for each replication cohort

Cohort	Variant	Chromosome:Position	Reference:Effect allele	Effect allele frequency	Odds ratio per allele	P-value
Australia	rs115261251	4:30696271	T:A	0.98	1.72	0.40
FinnGen	rs115261251	4:30696271	T:A	0.98	1.18	0.33
Italy	rs115261251	4:30696271	T:A	0.98	0.75	0.83
UK	rs115261251	4:30696271	T:A	0.98	0.78	0.60
USA	rs115261251	4:30696271	T:A	0.99	0.15	0.32
Australia	rs17245595	7:83604975	C:T	0.57	1.01	0.96
Brazil	rs17245595	7:83604975	C:T	0.56	1.04	0.93
FinnGen	rs17245595	7:83604975	C:T	0.58	0.89	**8.67×10^{-3}**
Italy	rs17245595	7:83604975	C:T	0.58	1.10	0.74
UK	rs17245595	7:83604975	C:T	0.56	1.11	0.25
USA	rs17245595	7:83604975	C:T	0.56	0.84	0.66
Australia	rs11984688	8:52647065	A:C	0.98	0.36	**3.43×10^{-3}**
FinnGen	rs11984688	8:52647065	A:C	0.99	1.51	**3.92×10^{-2}**
Italy	rs11984688	8:52647065	A:C	0.96	0.96	0.95
UK	rs11984688	8:52647065	A:C	0.98	0.51	**1.01×10^{-2}**
USA	rs11984688	8:52647065	A:C	0.97	0.65	0.57
Australia	rs746970	9:4217822	G:A	0.67	1.23	0.13
Brazil	rs746970	9:4217822	G:A	0.66	1.30	0.57
FinnGen	rs746970	9:4217822	G:A	0.71	0.86	**2.83×10^{-3}**
Italy	rs746970	9:4217822	G:A	0.71	0.91	0.76
UK	rs746970	9:4217822	G:A	0.66	1.26	**1.56×10^{-2}**
USA	rs746970	9:4217822	G:A	0.67	1.85	0.18
Australia	rs12913832	15:28365618	G:A	0.23	1.33	5.24×10^{-2}
Brazil	rs12913832	15:28365618	G:A	0.59	1.13	0.82
FinnGen	rs12913832	15:28365618	G:A	0.11	0.74	**7.73×10^{-6}**
Italy	rs12913832	15:28365618	G:A	0.47	0.86	0.63
UK	rs12913832	15:28365618	G:A	0.22	1.37	**2.26×10^{-3}**
USA	rs12913832	15:28365618	G:A	0.27	2.32	0.11

Associations of variants representing loci from the discovery conditional analysis by each replication cohort. Variant data are missing from Brazilian replication cohort for rs115261251 and rs11984688.

Supplementary Table 3: Lead variants from the multi-ancestry meta-analysis of 21 European and Asian cohorts (9217 PACG cases, 788,285 controls); comparison with European and Asian results

Variant	Chromosome: Position	Nearest Gene	Reference: Effect allele	Effect allele frequency	Odds ratio per allele	Multi-ancestry P-value	European ancestry P-value	Asian ancestry P-value	Novel locus
rs4908274	1:103389914	COL11A1	A:T	0.70	0.85	7.91×10^{-17}	4.71×10^{-7}	1.05×10^{-11}	
rs114976176	2:264621	SH3YL1	A:C	0.33	1.13	3.61×10^{-8}	1.54×10^{-3}	5.74×10^{-6}	✓
rs9877579	3:188058716	LPP	T:C	0.30	0.88	7.46×10^{-11}	1.38×10^{-5}	1.24×10^{-6}	✓
rs9873743	3:181349392	SOX2-OT	A:T	0.61	0.90	4.20×10^{-9}	8.48×10^{-6}	1.06×10^{-4}	✓
rs2914589	5:121266311	SRFBP1	T:C	0.14	1.20	9.55×10^{-9}	1.18×10^{-6}	2.23×10^{-3}	✓
rs11954540	5:143167638	HMHB1	T:G	0.79	0.88	3.78×10^{-8}	3.02×10^{-3}	1.24×10^{-6}	✓
rs4374796	6:73638262	KCNQ5	A:C	0.38	1.13	7.87×10^{-10}	3.34×10^{-7}	5.11×10^{-4}	
rs3816415	7:37988311	EPDR1	A:G	0.83	0.82	1.91×10^{-14}	5.35×10^{-6}	3.71×10^{-10}	
rs11760363	7:33179557	BBS9	T:C	0.12	0.79	1.52×10^{-12}	3.18×10^{-1}	1.68×10^{-22}	✓
rs6976211	7:83651854	SEMA3A	T:C	0.42	0.87	5.12×10^{-12}	2.41×10^{-8}	4.71×10^{-5}	
rs56277941	8:52898581	PCMTD1	T:G	0.14	1.53	4.98×10^{-14}	1.63×10^{-7}	2.75×10^{-8}	✓
rs746970	9:4217822	GLIS3	A:G	0.26	0.83	1.39×10^{-20}	7.22×10^{-12}	3.09×10^{-10}	
rs7869576	9:130768754	FAM102A	C:G	0.75	1.22	2.27×10^{-12}	1.53×10^{-1}	1.91×10^{-12}	
rs6478623	9:126315123	DENND1A	T:G	0.24	0.89	3.91×10^{-9}	6.66×10^{-8}	3.70×10^{-3}	
rs34927905	11:17001427	PLEKHA7	T:C	0.76	0.86	9.81×10^{-15}	2.63×10^{-4}	9.72×10^{-13}	
rs4938799	11:120208257	ARHGEF12	T:G	0.31	1.12	5.28×10^{-10}	2.69×10^{-4}	2.24×10^{-7}	✓
rs7494379	14:53411391	FERMT2	T:C	0.69	1.13	6.83×10^{-11}	1.67×10^{-5}	9.30×10^{-7}	
rs4151408	14:61434777	MNAT1	A:G	0.78	0.88	9.92×10^{-9}	8.13×10^{-4}	1.04×10^{-6}	✓
rs11665732	19:2047688	MKNK2	A:C	0.52	0.89	4.32×10^{-9}	9.23×10^{-5}	1.19×10^{-5}	✓
rs8136485	22:32871227	FBXO7	T:C	0.75	0.87	1.48×10^{-9}	5.48×10^{-6}	6.70×10^{-5}	✓
rs134545	22:28799080	TTC28	T:C	0.25	0.90	3.42×10^{-8}	3.91×10^{-5}	2.30×10^{-4}	✓

All P-values from the multi-ancestry meta-analysis are genome-wide significant ($P < 5 \times 10^{-8}$). Ticks against 'Novel locus' represents genome-wide significant variants in the multi-ancestry analysis which were not genome-wide significant in the European ancestry meta-analysis or previously reported.

Supplementary Table 4: Lead variants from the multi-ancestry meta-analysis of 21 European and Asian cohorts (9217 PACG cases, 788,285 controls); reported associations with refraction or iris morphology

Variant	Chromosome: Position	Nearest Gene	Ocular biometry or refraction (P-value)	Iris colour or morphology (P-value)	Any form of Glaucoma (P-value)
rs4908274	1:103389914	COL11A1	NEALE2_2217_raw (2.12×10^{-3})	NEALE2_1747_1 (6.55×10^{-5})	NEALE2_20002_1277 (2.13×10^{-7})
rs114976176	2:264621	SH3YL1	NEALE2_5084_raw (1.72×10^{-5})		
rs9877579	3:188058716	LPP			GCST009722 (5.13×10^{-10})
rs9873743	3:181349392	SOX2-OT	NEALE2_2207 (2.37×10^{-8})		FINNGEN_R6_H7_GLAUCPRIMOPEN (6.13×10^{-4})
rs2914589	5:121266311	SRFBP1		NEALE2_1747_4 (2.26×10^{-3})	GCST009722 (4.19×10^{-4})
rs11954540	5:143167638	HMHB1			
rs4374796	6:73638262	KCNQ5	NEALE2_5084_raw (1.19×10^{-53})		
rs3816415	7:37988311	EPDR1			GCST009722 (2.61×10^{-3})
rs11760363	7:33179557	BBS9			SAIGE_365_11 (1.00×10^{-3})
rs6976211	7:83651854	SEMA3A			SAIGE_365 (7.82×10^{-5})
rs56277941	8:52898581	PCMTD1			
rs746970	9:4217822	GLIS3			GCST009722 (6.17×10^{-7})
rs7869576	9:130768754	FAM102A			
rs6478623	9:126315123	DENND1A	NEALE2_5085_raw (4.86×10^{-3})	NEALE2_1747_1 (6.56×10^{-4})	
rs34927905	11:17001427	PLEKHA7			GCST009722 (8.68×10^{-15})
rs4938799	11:120208257	ARHGEF12			GCST009722 (7.06×10^{-15})
rs7494379	14:53411391	FERMT2			GCST009722 (1.06×10^{-11})
rs4151408	14:61434777	MNAT1			
rs11665732	19:2047688	MKNK2			
rs8136485	22:32871227	FBXO7	NEALE2_2217_raw (8.53×10^{-6})		FINNGEN_R6_H7_GLAUCCLOSEPRIM (1.27×10^{-5})
rs134545	22:28799080	TTC28			GCST009722 (3.17×10^{-11})

All P-values from the multi-ancestry meta-analysis are genome-wide significant ($P < 5 \times 10^{-8}$). Study identifiers and P-values are shown for variants having an association with ocular biometry or refraction, iris colour or morphology, and glaucoma, as reported by Open Targets Genetics [1].

Supplementary Table 5: Genetic correlation analyses with traits related to anthropometry, education, glaucoma, pigmentation and refraction, in Europeans

Category	Trait	Reference	Genetic correlation	Standard error	Z-Score	P-value
Anthropometry	Standing height	MRC-IEU: ukb-b-10787	0.00	0.03	0.03	9.78×10^{-1}
Anthropometry	Waist circumference	MRC-IEU: ukb-b-9405	-0.04	0.03	-1.24	2.17×10^{-1}
Education	Educational attainment	PMID: 33414549	-0.03	0.04	-0.75	4.52×10^{-1}
Glaucoma-related	Intraocular pressure	PMID: 29785010	-0.14	0.05	-3.10	**1.96×10^{-3}**
Glaucoma-related	Vertical cup to disc ratio	PMID: 34077760	-0.08	0.05	-1.67	9.50×10^{-2}
Glaucoma-related	Macular Retinal nerve fibre layer	PMID: 33979322	-0.10	0.07	-1.50	1.33×10^{-1}
Glaucoma-related	Ganglion cell–inner plexiform layer	PMID: 33979322	0.12	0.06	1.95	5.18×10^{-2}
Pigmentation	Darker Skin colour	MRC-IEU: ukb-b-19560	-0.01	0.04	-0.12	9.01×10^{-1}
Pigmentation	Black hair colour	MRC-IEU: ukb-d-1747_5	0.01	0.05	0.19	8.47×10^{-1}
Refraction	Axial length	PMID: 37351342	-0.50	0.09	-5.75	**9.04×10^{-9}**
Refraction	Refractive error	PMID: 32231278	-0.41	0.04	-10.90	**1.21×10^{-27}**
Refraction	Hypermetropia	MRC-IEU: ukb-b-18189	0.45	0.11	4.18	**2.94×10^{-5}**
Refraction	Age when started wearing glasses	MRC-IEU: ukb-b-5801	0.24	0.05	5.09	**3.58×10^{-7}**
Refraction	Cataracts	PMID: 34127677	-0.17	0.05	-3.10	**1.91×10^{-3}**

Genetic correlation of the largest publicly available summary statistics for a set of traits using linkage disequilibrium score regression. Traits are grouped into categories: glaucoma-related, refraction, pigmentation, education and anthropometry. P-value < 0.05 in bold.

Supplementary Table 6: Two-sample Mendelian randomisation for axial length, eye colour and refractive error on PACG

Method	Axial length (mm, 13 SNPs)		Eye colour (Z-score, 41 SNPs)		Refractive error (Z-score, 377 SNPs)	
	OR (95% CI)	P-value	OR (95% CI)	P-value	OR (95% CI)	P-value
IVW	0.34 (0.29,0.40)	6.22×10^{-41}	1.10 (1.06,1.14)	4.73×10^{-7}	2.99 (2.58,3.47)	2.02×10^{-49}
Weighted median	0.36 (0.28,0.46)	1.07×10^{-14}	1.10 (1.08,1.13)	4.25×10^{-17}	3.76 (3.03,4.66)	1.18×10^{-36}
Weighted mode	0.38 (0.26,0.55)	7.39×10^{-4}	1.11 (1.08,1.13)	7.81×10^{-10}	4.49 (3.33,6.06)	8.17×10^{-21}
MR-Egger	0.16 (0.07,0.38)	1.64×10^{-3}	1.10 (1.06,1.15)	2.19×10^{-5}	5.16 (3.78,7.04)	4.88×10^{-23}
MR-PRESSO	0.34 (0.29,0.40)	6.22×10^{-41}	1.10 (1.06,1.14)	4.73×10^{-7}	3.06 (2.65,3.52)	6.93×10^{-44}

Mendelian randomisation (MR) analysis was performed for each of the exposures (axial length (mm), eye colour (Z-score) and refractive error (Z-score)) and the outcome (PACG, using the summary statistics from the European ancestry meta-analysis). Instrumental variables for exposures were independent variants at genome-wide significance from the largest available genome-wide association studies in Europeans. Odds ratios expressed per additional allele. Exposure summary statistics for axial length were from the GERA cohort [2], eye colour from 23andMe and the VisiGen Consortium [3] and refractive error from UK Biobank [4].

Supplementary Table 7: Logistic regression models of PACG and PAC cases and controls in EPIC-Norfolk

term	Model-1		Model-2		Model-3		Model-4	
	OR (95% CI)	P-value	OR (95% CI)	P-value	OR (95% CI)	P-value	OR (95% CI)	P-value
PRS-A (standardised)					1.92 (1.44-2.57)	**8.89×10^{-6}**	1.79 (1.32-2.42)	**1.83×10^{-4}**
SE (dioptre)			1.24 (1.07-1.44)	**4.77×10^{-3}**			1.13 (0.97-1.32)	0.12
Age	1.06 (1.03-1.10)	**8.74×10^{-4}**	1.06 (1.02-1.10)	**3.20×10^{-3}**	1.06 (1.02-1.10)	**9.32×10^{-4}**	1.06 (1.02-1.10)	**1.66×10^{-3}**
Sex	1.62 (0.90-3.01)	0.12	1.60 (0.89-2.98)	0.13	1.65 (0.91-3.07)	0.10	1.65 (0.91-3.07)	0.10

Odds ratio (OR) per allele (95% confidence intervals (CI)) are shown for logistic regression models: Model-1, age and sex; Model-2 age, sex and spherical equivalent (SE, dioptre); Model-3 PRS-A, age and sex; Model-4 PRS-A, SE, age and sex. Variance explained (pseudo R^2) for each model was: Model-1 0.023, Model-2 0.038, Model-3 0.059, Model-4 0.063. All PACG-related traits measured in the EPIC-Norfolk Eye Study. PACG and primary angle closure (PAC) cases are defined clinically.

Supplementary Table 8: The association of PACG-related traits in EPIC-Norfolk with quintiles of polygenic risk score derived from the PACG European ancestry meta-analysis

Phenotype	Continuous (Standardised) Effect (95% CI)	Quintile 1	Quintile 2 Effect (95% CI)	Quintile 3 Effect (95% CI)	Quintile 4 Effect (95% CI)	Quintile 5 Effect (95% CI)	Trend (P-value)
Anterior chamber depth (mm)	-0.19(-0.23,-0.14)	Ref	**-0.04 (-0.07, 0.00)**	**-0.05 (-0.08,-0.02)**	**-0.09 (-0.13,-0.06)**	**-0.12(-0.16,-0.09)**	**4.28×10^{-14}**
Axial length (mm)	-0.17 (-0.21,-0.12)	Ref	-0.08 (-0.16, 0.01)	-0.08 (-0.17, 0.00)	**-0.18 (-0.26,-0.09)**	**-0.27(-0.36,-0.19)**	**3.55×10^{-11}**
Intraocular pressure (mmHg)	0.11 (0.06,0.16)	Ref	**0.32 (0.02,0.62)**	**0.43 (0.12,0.73)**	**0.31 (0.00,0.61)**	**0.66 (0.35,0.97)**	**1.83×10^{-4}**
Spherical equivalent (D)	0.09 (0.05,0.14)	Ref	0.07 (-0.09,0.24)	**0.23 (0.06,0.39)**	**0.31 (0.14,0.47)**	**0.30 (0.13,0.46)**	**9.92×10^{-6}**
Blue/grey eye colour vs other eye colours	—	Ref	0.87 (0.75,1.01)	**0.85 (0.74,0.99)**	**0.85 (0.74,0.99)**	**0.57 (0.50,0.67)**	**2.17×10^{-11}**
Primary open angle glaucoma vs controls	—	Ref	1.03 (0.84,1.25)	0.89 (0.73,1.08)	1.10 (0.90,1.35)	0.95 (0.78,1.15)	0.86

Beta (95% confidence intervals) are shown for linear regression models: axial length, anterior chamber depth and spherical equivalent regressed on quintiles of meta-analysis derived polygenic risk score (PRS-B), age and sex. Odds ratio per allele (95% confidence intervals) are shown for logistic regression models: blue or grey eye colour and primary open angle glaucoma (POAG) on PRS-B, age and sex. All PACG-related traits measured in the EPIC-Norfolk Eye Study. CI is confidence interval. Continuous associations used standardised variables with mean 0 and standard deviation 1. Figures in bold have P-value < 0.05.

Supplementary Table 9: Dominant and recessive models for GWAS significant loci from the European meta-analysis

Variant	Model	Odds ratio (95% CI) per allele	P-value
rs12913832	Additive: AA,AG,GG	0.77 (0.72,0.84)	4.01×10^{-10}
	Dominant: AA+AG ~ GG	0.80 (0.65,1.00)	0.046
	Recessive: AA ~ AG+GG	0.72 (0.65,0.79)	2.48×10^{-10}
rs73199928	Additive: AA,AG,GG	0.56 (0.45,0.71)	5.87×10^{-7}
	Dominant: AA+AG ~ GG	-	-
	Recessive: AA ~ AG+GG	0.55 (0.44,0.69)	2.24×10^{-7}
rs7744813	Additive: AA,AC,CC	1.17 (1.09,1.26)	1.79×10^{-5}
	Dominant: AA+AC ~ CC	1.22 (1.09,1.36)	4.49×10^{-4}
	Recessive: AA ~ AC+CC	1.18 (1.03,1.35)	0.016
rs12193446	Additive: AA,AG,GG	1.23 (1.10,1.37)	1.88×10^{-4}
	Dominant: AA+AG ~ GG	1.24 (1.10,1.40)	5.21×10^{-4}
	Recessive: AA ~ AG+GG	1.37 (0.87,2.04)	0.15
rs142455016	Additive: CC,CT,TT	2.35 (1.67,3.20)	2.64×10^{-7}
	Dominant: CC+CT ~ TT	2.31 (1.63,3.17)	7.51×10^{-7}
	Recessive: CC ~ CT+TT	8.93 (0.49,46.32)	0.037
rs10215636	Additive: CC,CT,TT	1.23 (1.14,1.33)	6.54×10^{-8}
	Dominant: CC+CT ~ TT	1.40 (1.19,1.65)	5.50×10^{-5}
	Recessive: CC ~ CT+TT	1.21 (1.09,1.34)	4.43×10^{-4}
rs11984688	Additive: AA,AC,CC	0.51 (0.42,0.63)	3.88×10^{-11}
	Dominant: AA+AC ~ CC	0.23 (0.07,1.40)	0.040
	Recessive: AA ~ AC+CC	0.52 (0.42,0.64)	2.70×10^{-10}
rs111736202	Additive: AA,AG,GG	0.60 (0.50,0.73)	1.94×10^{-7}
	Dominant: AA+AG ~ GG	0.12 (0.06,0.29)	2.06×10^{-7}
	Recessive: AA ~ AG+GG	0.65 (0.53,0.81)	5.62×10^{-5}
rs746970	Additive: AA,AG,GG	0.80 (0.74,0.87)	3.04×10^{-8}
	Dominant: AA+AG ~ GG	0.77 (0.70,0.85)	2.61×10^{-7}
	Recessive: AA ~ AG+GG	0.81 (0.67,0.97)	0.027
rs10818834	Additive: CC,CT,TT	1.18 (1.08,1.28)	1.37×10^{-4}
	Dominant: CC+CT ~ TT	1.03 (0.85,1.26)	0.76
	Recessive: CC ~ CT+TT	1.30 (1.17,1.45)	2.23×10^{-6}

Odds ratio per allele (95% confidence intervals) are shown for logistic regression additive, dominant and recessive models adjusted for age and sex. All PACG-related traits measured in the EPIC-Norfolk Eye Study. CI is confidence interval. The dominant model for rs73199928 did not converge.

Supplementary figures

Supplementary Figure 1: Forest plot presenting the associations with PACG for lead discovery GWAS variants in the discovery and replication cohorts

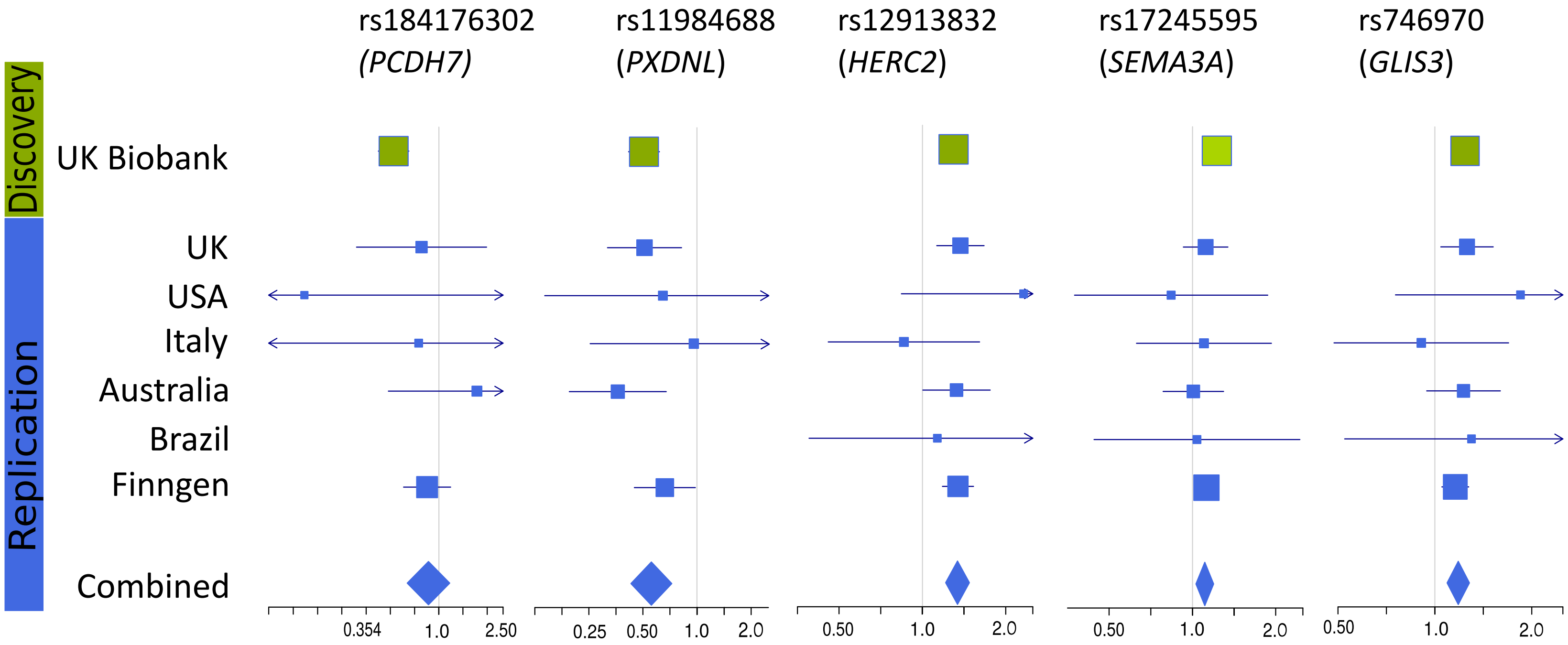

Size of squares correspond to size of cohort population. Lines show 95% confidence intervals. Diamonds represent meta-analysed replication study overall associations. The 'Combined' results reflects a fixed effects meta-analysis of the results for the six replication cohorts, and does not include the discovery (UK Biobank) cohort. Loci are labelled using the nearest genes to each locus. GWAS, genome-wide association study.

Supplementary Figure 2: Regional association analysis for PACG genome-wide significant loci from PACG European meta-analysis

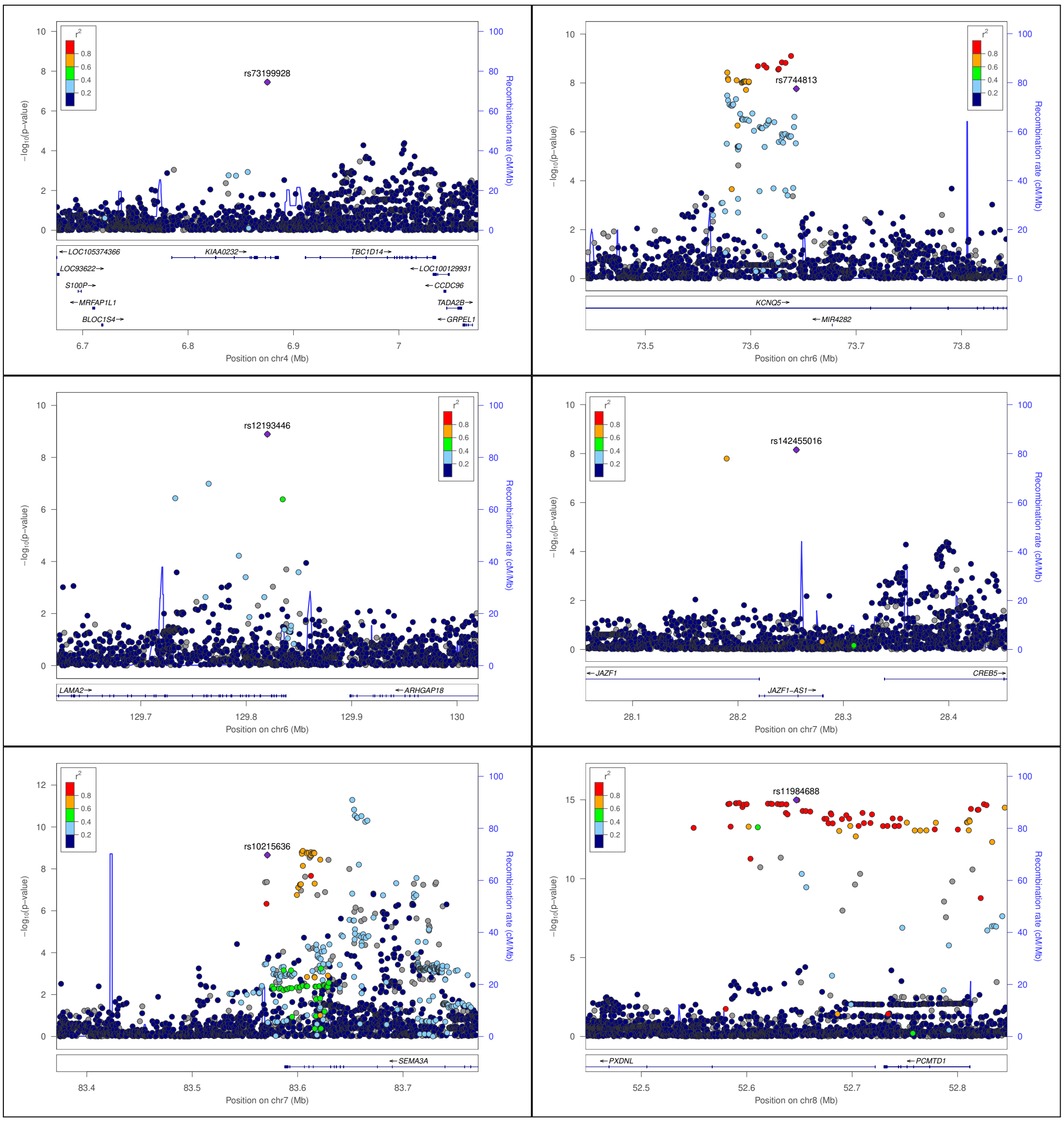

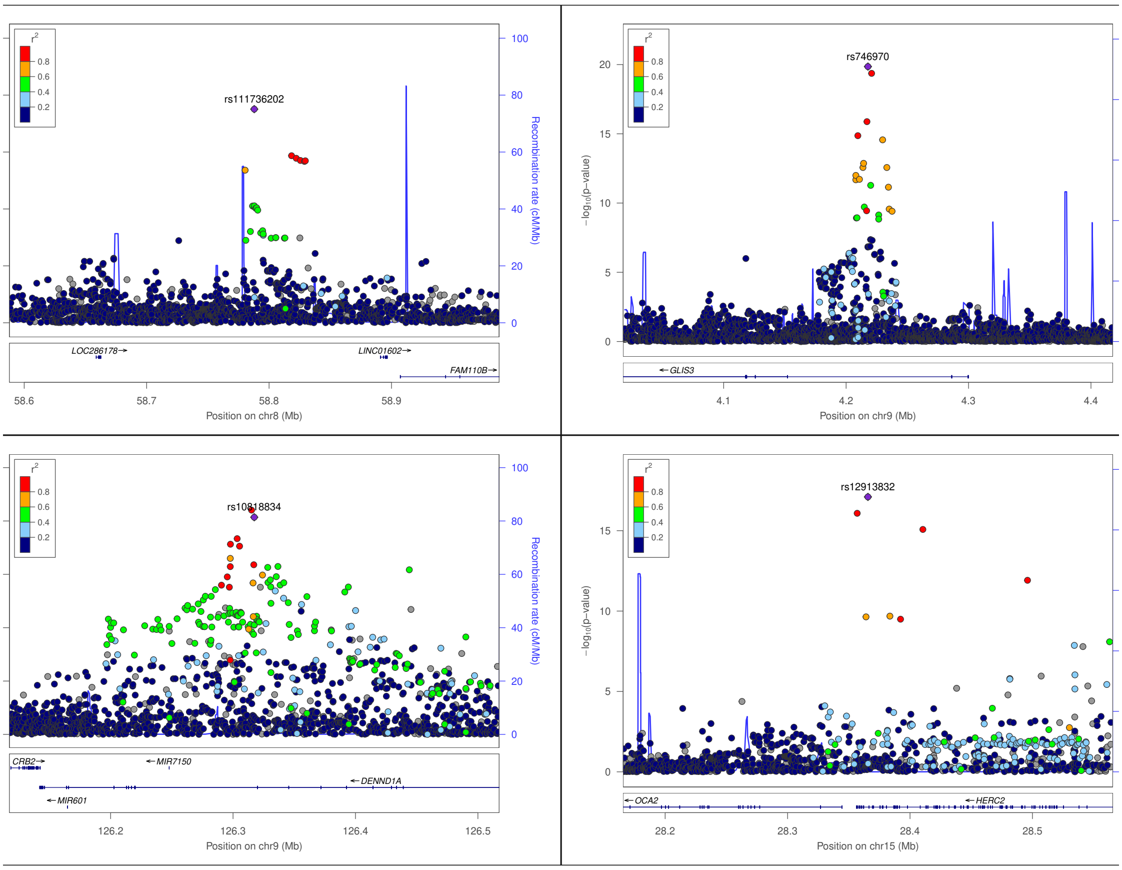

Association results for genome-wide significant loci in the European meta-analysis at ten locations near *KIAA0232* (rs73199928), *KCNQ5* (rs7744813), *LAMA2* (rs12193446), *JAZF1* (rs142455016), *SEMA3A* (rs10215636), *PXDNL* (rs11984688), *FAM110B* (rs111736202), *GLIS3* (rs746970), *DENND1A* (rs10818834) and *HERC2* (rs12913832). The graphs plot -log10 P-values on the y-axis against genome assembly GRCh37 on the x-axis. Round coloured markers illustrate the r-squared value for linkage disequilibrium with respect to the most significant variant of the locus marked by a purple diamond.

Supplementary Figure 3: Manhattan plot for GWAS of the European meta-analysis

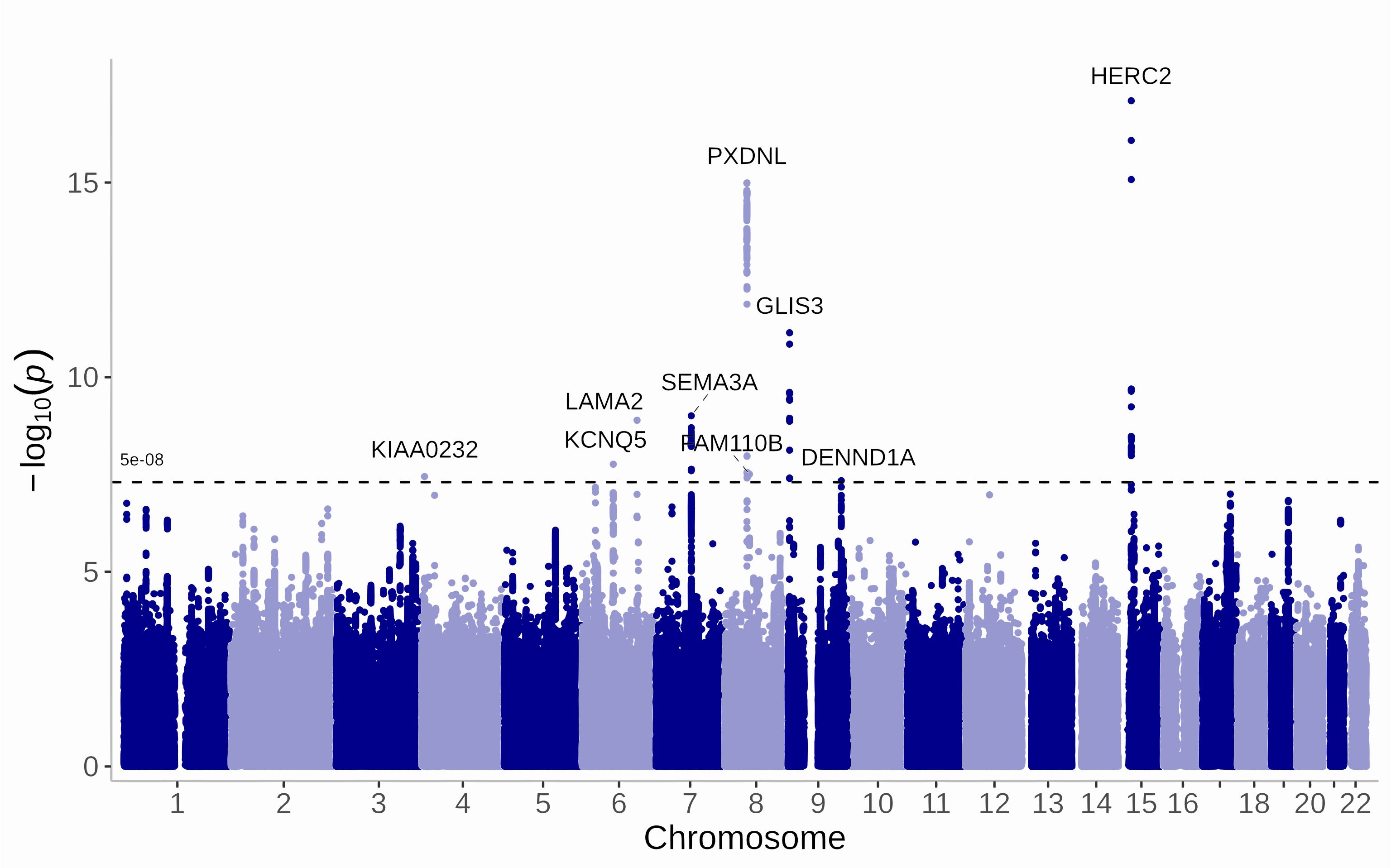

Manhattan plot using combined summary statistics from meta-analysed European discovery and replication cohorts of 3,183 PACG cases and 773,214 controls. GWAS associations used logistic regression with significance level $P < 5 \times 10^{-8}$ to account for multiple comparisons, and the Wald test statistic with two-sided testing. GWAS, genome-wide association study.

Supplementary Figure 4: Comparison of the effect size of genome-wide significant loci in the European meta-analyses with loci in the Asian meta-analyses

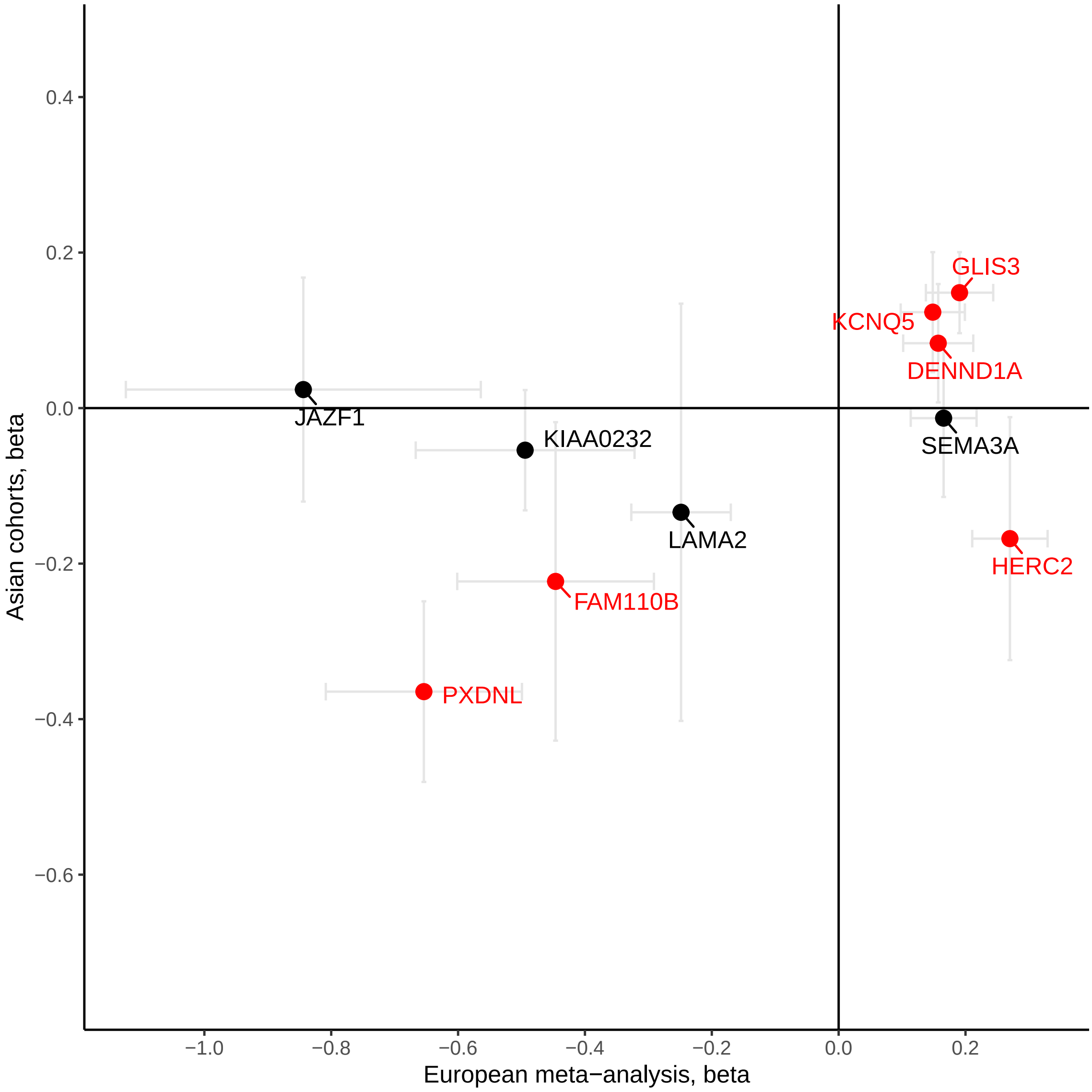

Effect size comparing the genome-wide significant loci in the European meta-analysis and published results of Asian populations [5]. All loci are genome-wide significant in Europeans; those significant at $P < 0.05$ in Asian data are represented by red points and non-significant by black points. Error bars represent 95% confidence intervals. Loci are labelled using their nearest gene. Loci are represented using a variant common to both datasets or proxy (group A in Supplementary Table 3).

Supplementary Figure 5: Multi-ancestry meta-analysis Manhattan plot of European and Asian cohorts

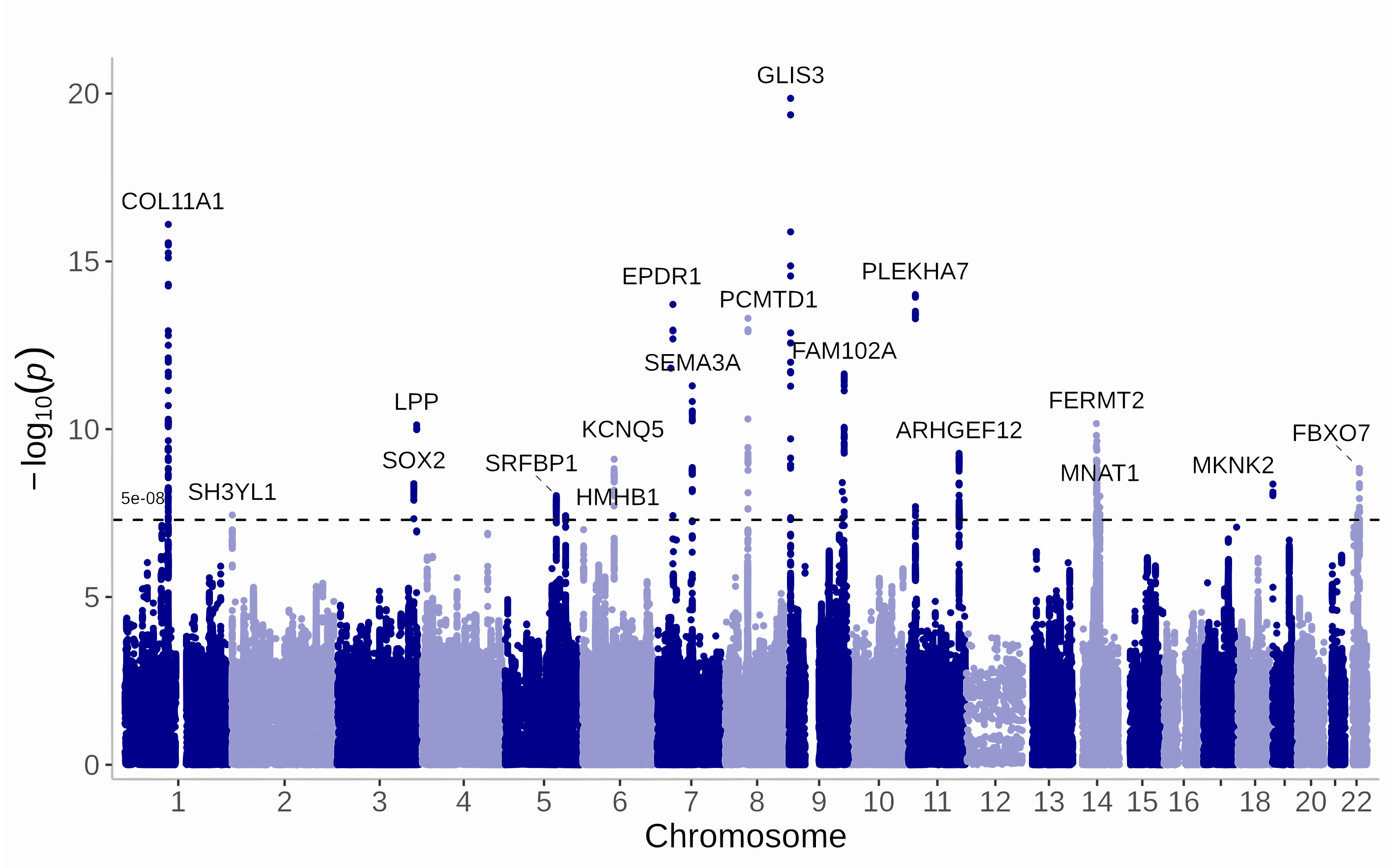

Manhattan plot using combined summary statistics from meta-analysed European discovery, 6 replication and 14 Asian cohorts. GWAS associations used logistic regression with significance level P < 5 x 10^{-8} to account for multiple comparisons, and the Wald test statistic with two-sided testing. GWAS, genome-wide association study.

Supplementary Figure 6: Genetic correlations of PACG-related traits with PACG

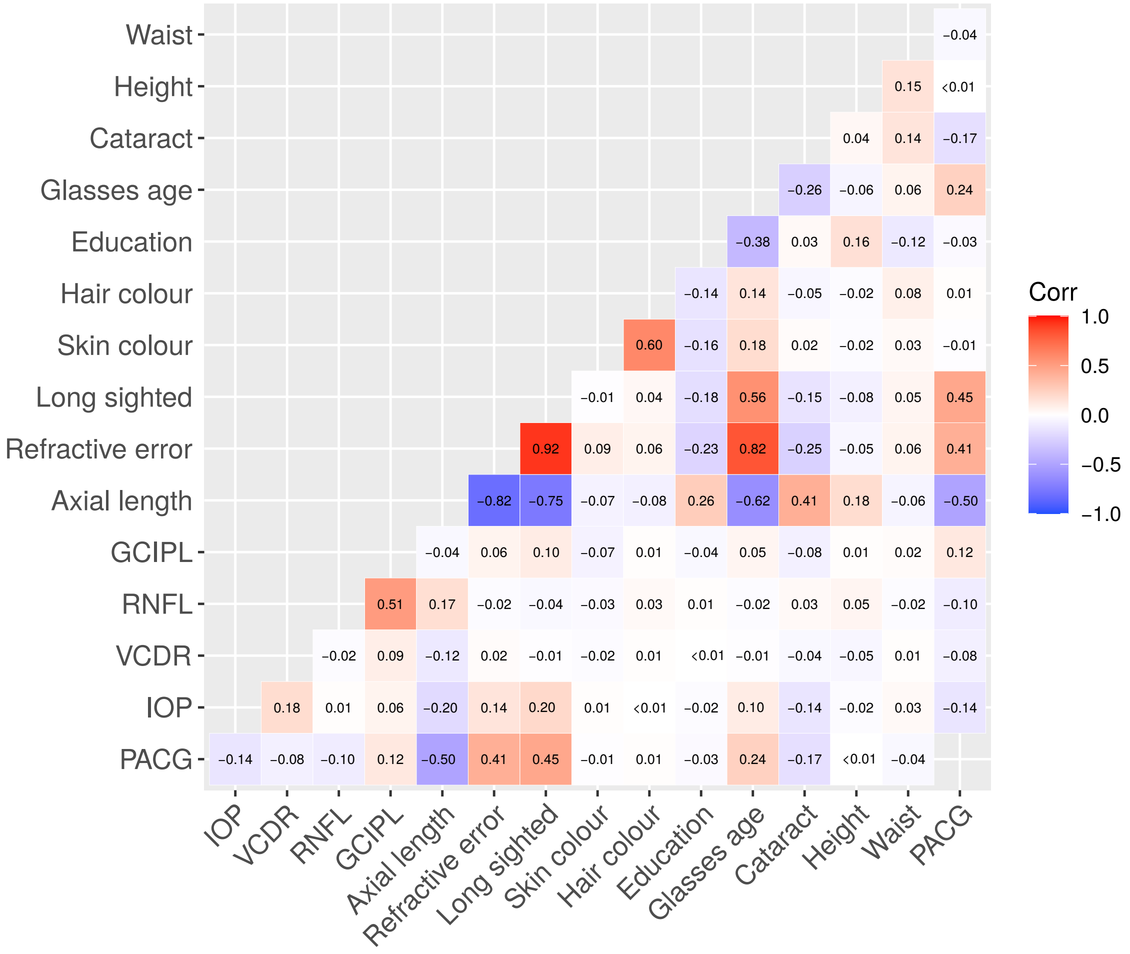

Abbreviations used in genetic correlation labels: PACG,PACG European Meta-analysis; Waist, Waist circumference; Height,Standing height; Cataract,Cataracts; Glasses age, Age when started wearing glasses; Education, Educational attainment; Hair colour,Hair colour; Skin colour, Skin colour; Long sighted, Hypermetropia / Long sightedness; Refractive error, Refractive error; Axial length, Axial length; GCIPL, Ganglion cell–inner plexiform layer; RNFL, Retinal nerve fibre layer; VCDR, Vertical cup to disc ratio; IOP, Intraocular pressure.

Supplementary Figure 7: Results for Mendelian randomisation experiment examining the potential causal effect of refractive error on PACG

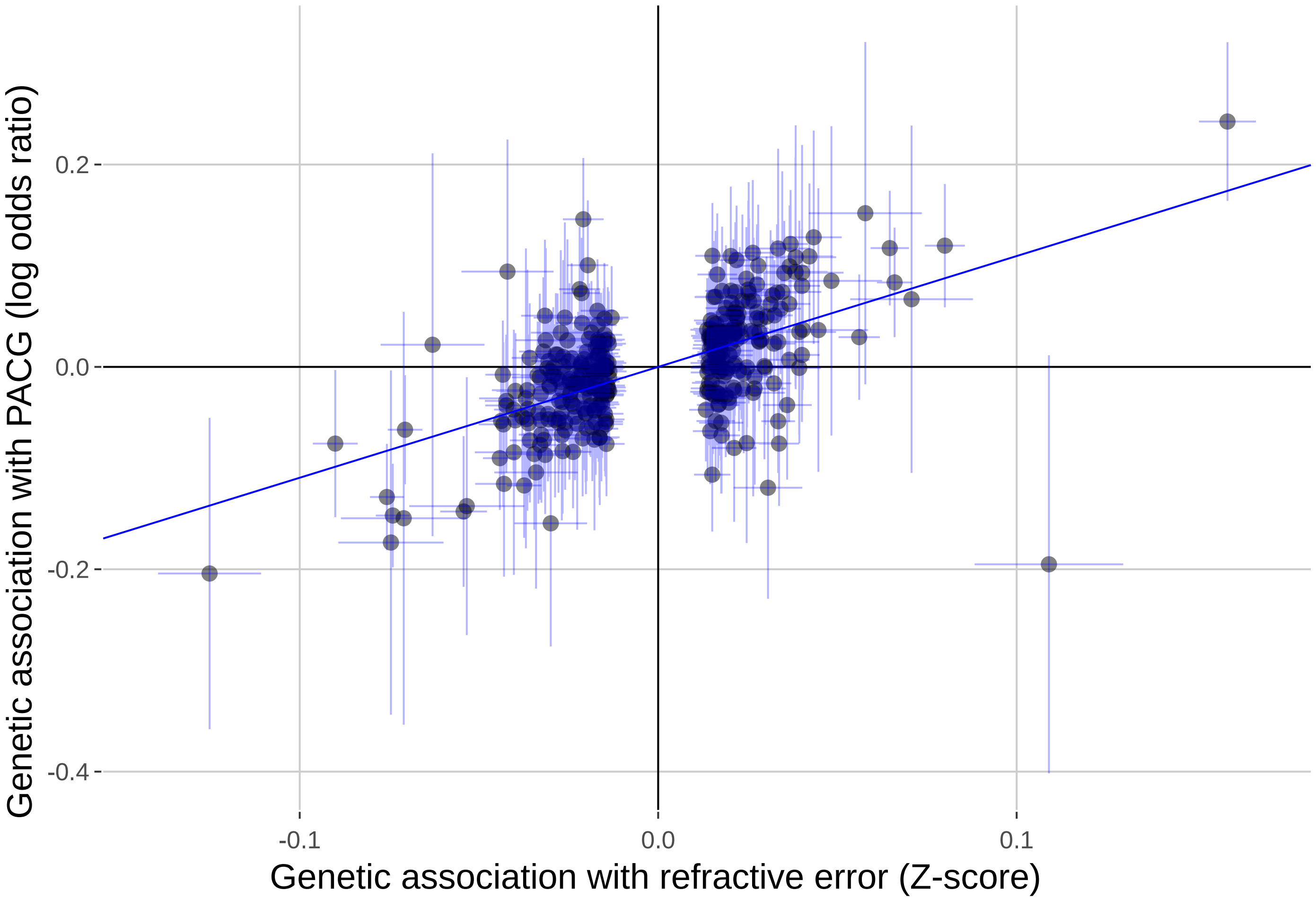

Two-sample inverse-variance weighted Mendelian randomisation using 377 independent genome-wide significant refractive error loci as exposure instrumental variables and European meta-analysis PACG summary statistics as outcome. Data are represented as log odds ratios for genetic associations of PACG and corresponding genetic association of refractive error Z-score for each loci. Error bars represent 95% confidence intervals. All units are per risk allele.

Supplementary Figure 8: Results for Mendelian randomisation experiment examining the potential causal effect of eye colour on PACG

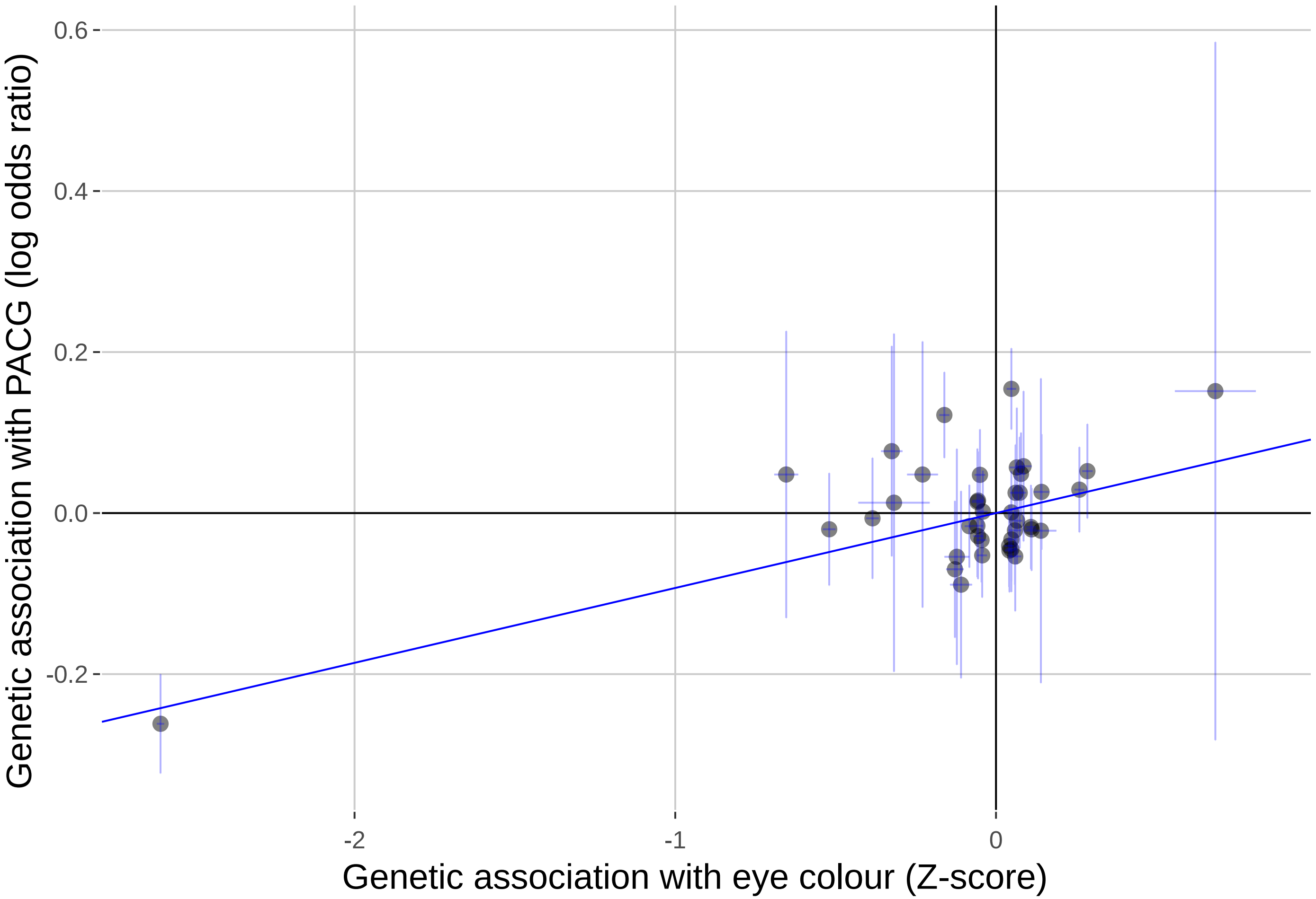

Two-sample inverse-variance weighted Mendelian randomisation using 41 independent genome-wide significant eye colour loci as exposure instrumental variables and European meta-analysis PACG summary statistics as outcome. Data are represented as log odds ratios for genetic associations of PACG and corresponding genetic association of eye colour Z-score for each loci. Error bars represent 95% confidence intervals. All units are per risk allele.

Supplementary Figure 9: Two-sample Mendelian randomisation leave-one-out analysis of eye colour loci and PACG

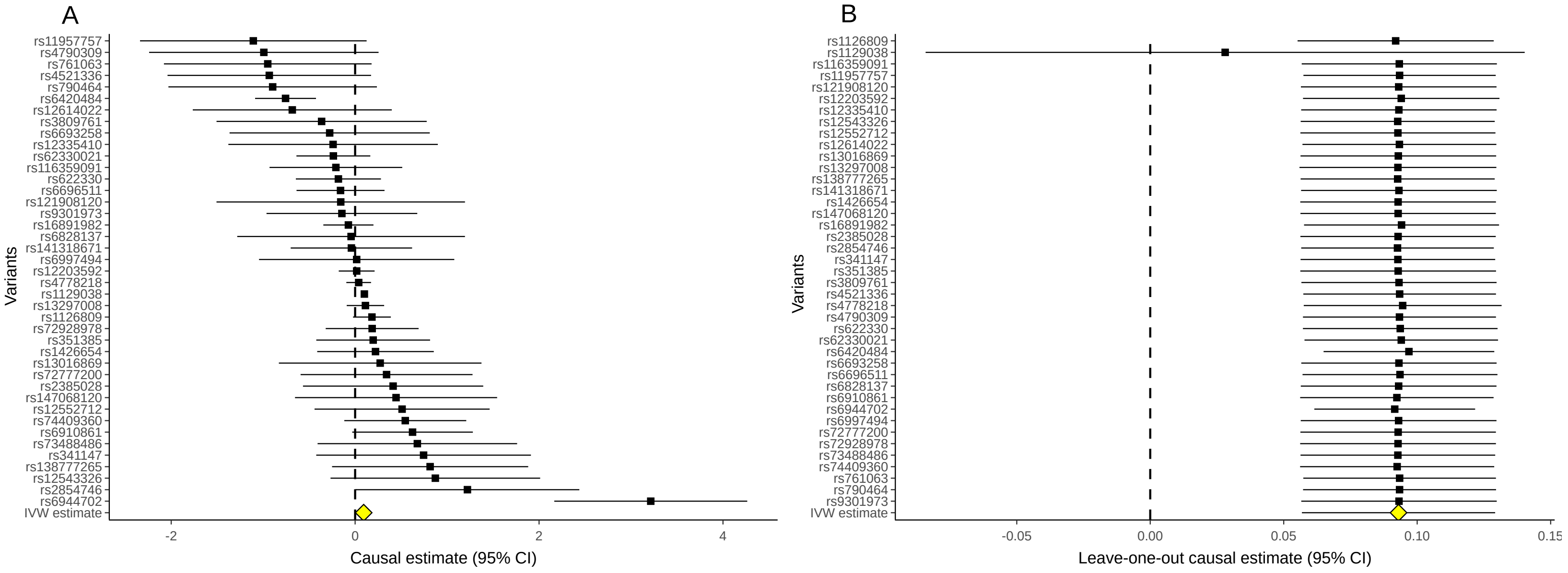

A Causal estimates for 41 independent genome-wide significant eye colour loci in the MR analysis of eye colour and PACG. **B** Leave-one-out analysis identifying one strong effect variant (rs1129038 in the region of *HERC2* and *OCA2*).

Supplementary Figure 10: Polygenic risk score PRS-A from the MTAG analysis adjusted for spherical equivalent applied to 47 PACG and PAC cases and 6,623 controls in the EPIC-Norfolk study

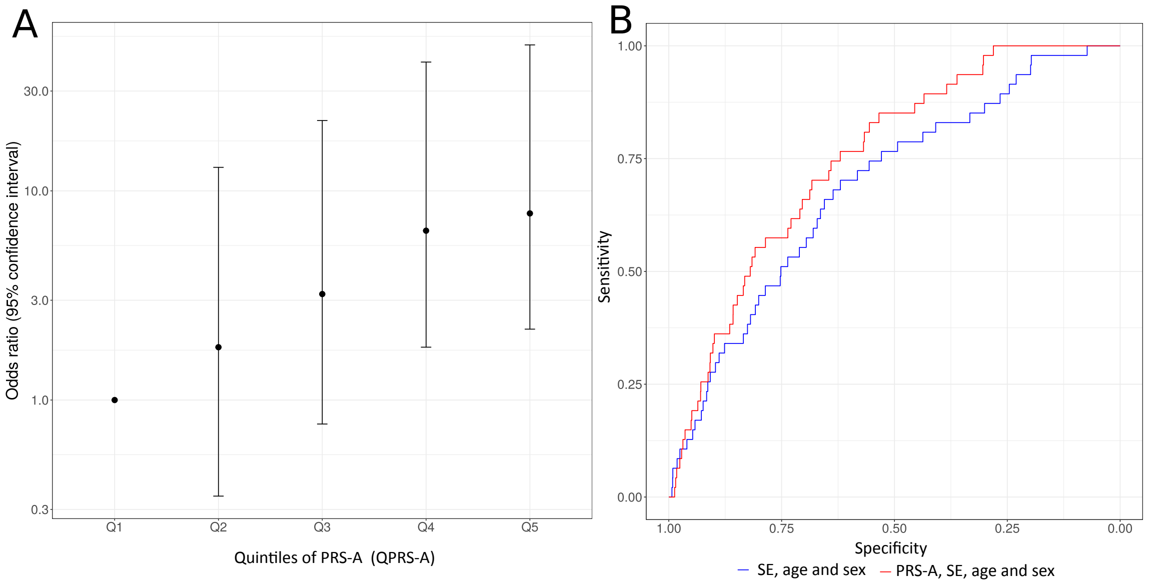

Summary statistics

Logistic regression models

PACG/PAC ~ PRS-A + SE + age + sex: $P = 1.83 \times 10^{-4}$

PACG/PAC ~ QPRS-A + SE + age + sex: $P_{trend} = 9.89 \times 10^{-5}$

Q1 vs Q5: OR (95% CI) = 7.80 (2.18–49.9)

Receiver operating characteristic curve (plot B)

PACG/PAC ~ SE + age + sex: AUROC = 0.69

PACG/PAC ~ PRS-A + SE + age + sex: AUROC = 0.75

DeLong test for difference: *P* = 0.023

A Odds ratio of PACG and primary angle closure (PAC) cases versus controls by quintiles of multi-trait analysis of genome-wide association study (MTAG) polygenic risk score A (PRS-A) adjusted for SE, age and sex with quintile 1 as reference. Error bars represent 95% confidence intervals. **B** Receiver operating characteristic (ROC) curves comparing models for PRS-A, spherical equivalent (SE), age and sex (red) and for SE, age and sex (blue). PACG is defined clinically. Controls exclude other forms of glaucoma and suspected glaucoma. QPRS-A, quintiles of PRS-A.

Supplementary Figure 11: Plots of PACG-related traits in EPIC-Norfolk with quintiles of polygenic risk score PRS-B derived from the PACG European ancestry meta-analysis

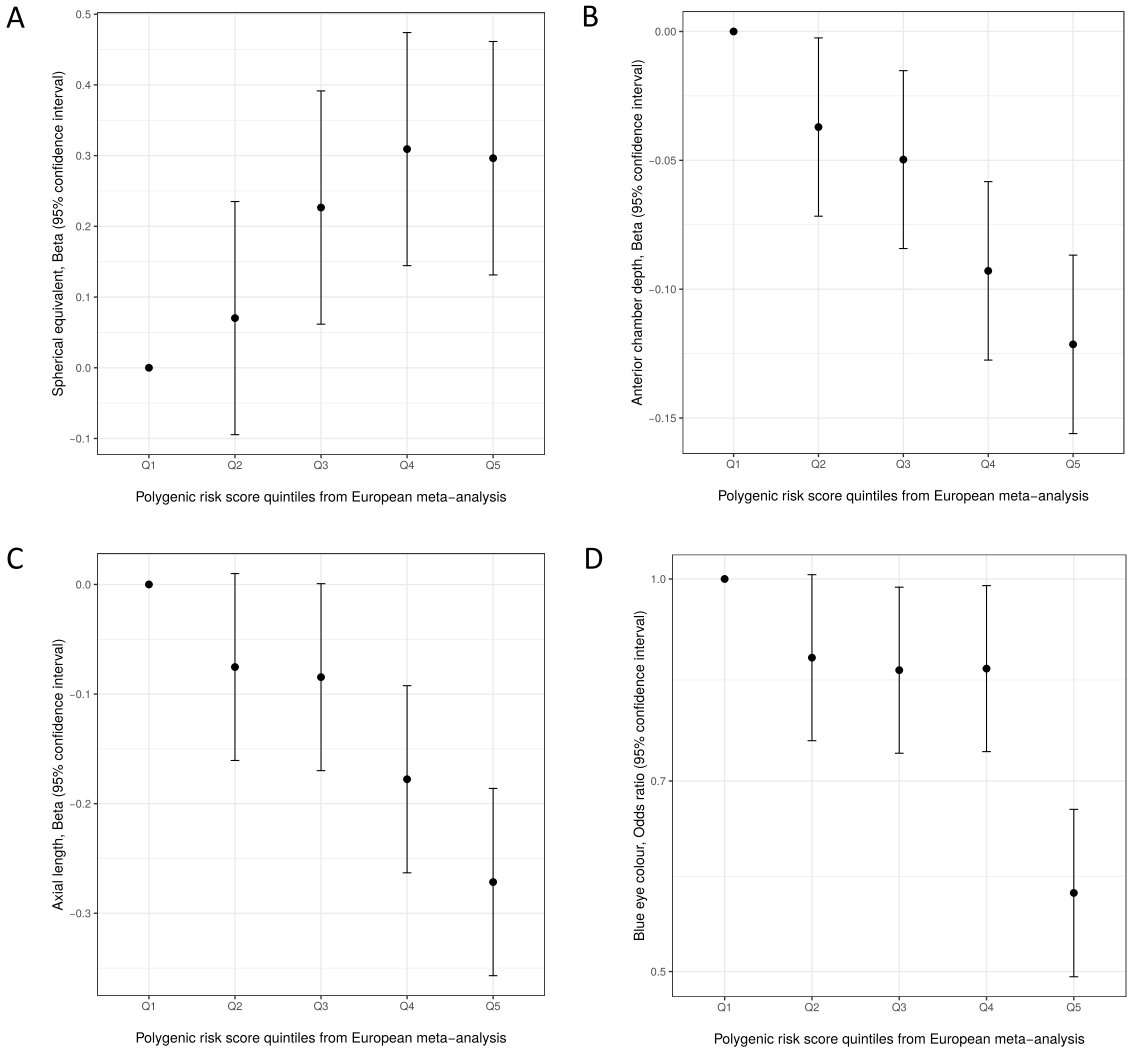

Associations of European ancestry meta-analysis polygenic risk score B (PRS-B) in quintiles and **A** spherical equivalent, **B** axial length, **C** anterior chamber depth and **D** blue or grey eyes. All models were adjusted for age and sex. Data are presented as odds ratios and error bars represent 95% confidence intervals. PRS-B was available for 7,223 EPIC-Norfolk participants. Trait measurements were available for: spherical equivalent (7,129); axial length (6,616); anterior chamber depth (6,530); eye colour (7,223).

Supplementary Figure 12: Polygenic risk score PRS-B from the European meta-analysis applied to 47 PACG and PAC cases and 6,623 controls in the EPIC-Norfolk study

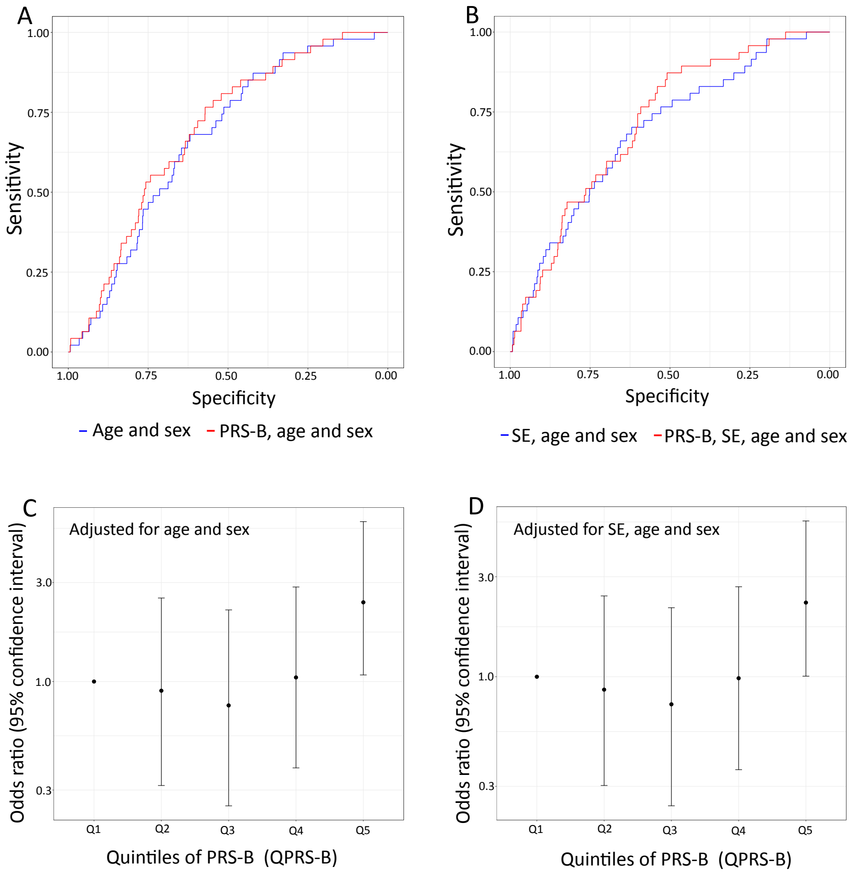

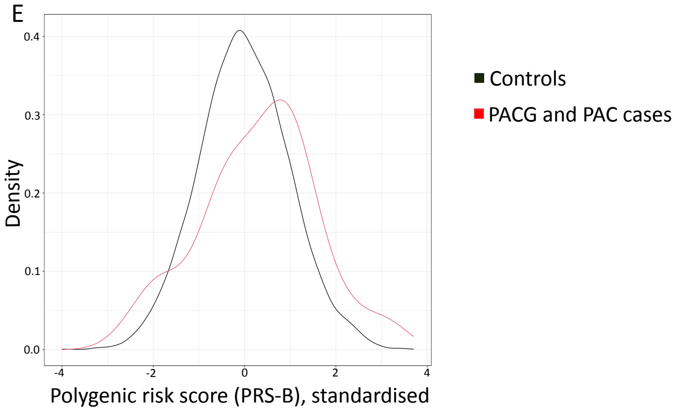

Receiver operating characteristic curve

PACG/PAC ~ age + sex: AUROC = 0.67 (Plot A)
PACG/PAC ~ PRS-B + age + sex: AUROC = 0.69 (Plot A)
DeLong test for difference: *P* = 0.43 (Plot A)

PACG/PAC ~ SE + age + sex: AUROC = 0.69 (Plot B)
PACG/PAC ~ PRS-B + SE+ age + sex: AUROC = 0.71 (Plot B)
DeLong test for difference: *P* = 0.26 (Plot B)

Logistic regression models

PACG/PAC ~ PRS-B + age + sex: *P* = 0.025
PACG/PAC ~ QPRS-B + age + sex: P_{trend} = 0.023 (Plot C)
Q1 vs Q5: OR (95% CI) = 2.41 (1.08–5.90) (Plot C)

PACG/PAC ~ PRS-B + SE + age + sex: *P* = 0.036
PACG/PAC ~ QPRS-B + SE + age + sex: P_{trend} = 0.033 (Plot D)
Q1 vs Q5: OR (95% CI) = 2.26 (1.01–5.54) (Plot D)

A Receiver operating characteristic (ROC) curves comparing models for PRS-B, age and sex (red) and for age and sex (blue). **B** Receiver operating characteristic (ROC) curves comparing models for PRS-B, SE, age and sex (red) and for SE, age, sex (blue). **C** Odds ratio of PACG and PAC cases versus controls by quintiles of European meta-analysis PRS-B adjusted for age and sex with quintile 1 as reference. Error bars represent 95% confidence intervals. **D** Odds ratio of PACG and PAC cases versus controls by quintiles of European meta-analysis PRS-B adjusted for SE, age and sex with quintile 1 as reference. Error bars represent 95% confidence intervals. **E** Density plots of standardised polygenic risk score B (PRS-B) for PACG and primary angle closure (PAC) cases (red) and controls (black). PACG is defined clinically. Controls exclude other forms of glaucoma and suspected glaucoma. QPRS-B, quintiles of PRS-B.

Supplementary Figure 13: Polygenic risk score PRS-A from the MTAG analysis applied to 137 PACG and APAC cases and 245 controls from replication cohort participants with non-European ancestry

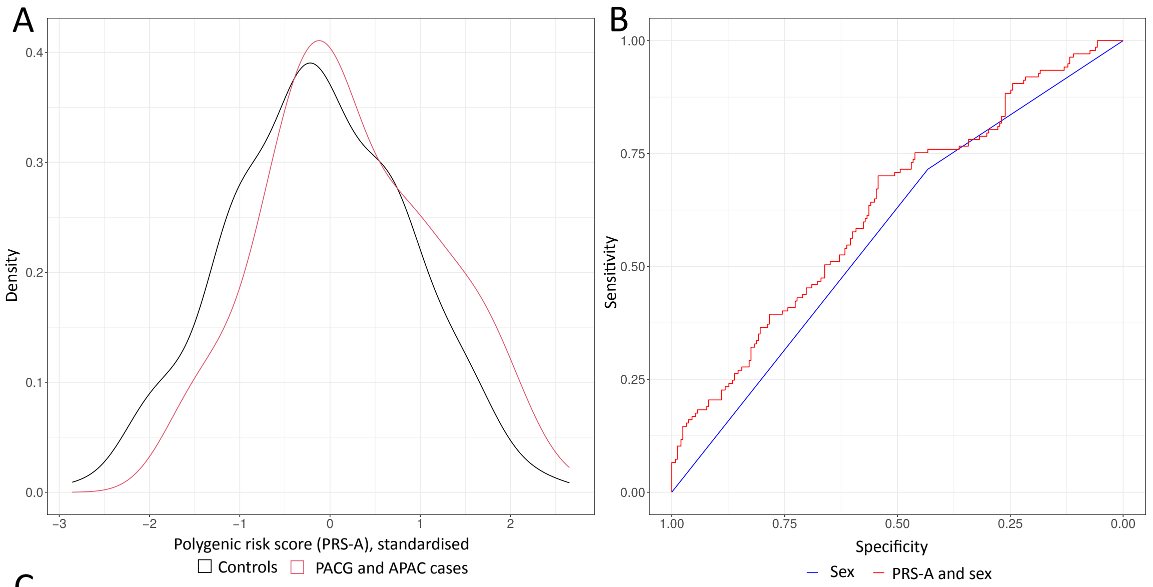

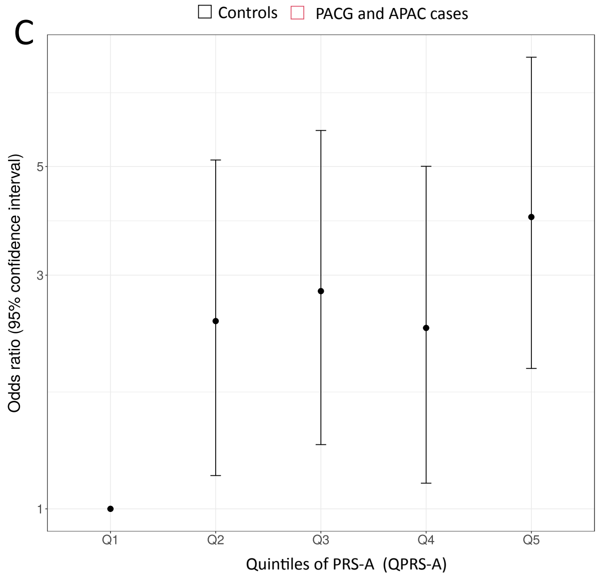

Summary statistics

Logistic regression models

PACG/PAC ~ PRS-A + age + sex: $P = 6.91 \times 10^{-4}$
PACG/PAC ~ QPRS-A + age + sex: $P_{trend} = 1.07 \times 10^{-3}$
Q1 vs Q5: OR (95% CI) = 3.94 (1.93–8.36)

Receiver operating characteristic curve (plot B)

PACG/PAC ~ age + sex: AUROC = 0.57
PACG/PAC ~ PRS-A + age + sex: AUROC = 0.63
DeLong test for difference: $P = 1.66 \times 10^{-2}$

A Density plots of standardised polygenic risk score A (PRS-A) for PACG and acute primary angle closure (APAC) cases (red) and controls (black). **B** Receiver operating characteristic (ROC) curves comparing models for PRS-A, spherical equivalent (SE), age and sex (red) and for SE, age and sex (blue). **C** Odds ratio of PACG and APAC cases versus controls by quintiles of multi-trait analysis of genome-wide association study (MTAG) PRS-A adjusted for SE, age and sex with quintile 1 as reference. Error bars represent 95% confidence intervals. PACG and APAC are defined clinically in participants from cohorts in Australia, Brasil, Italy, UK and USA with non-European ancestry. Controls exclude other forms of glaucoma and suspected glaucoma. QPRS-A, quintiles of PRS-A.

Supplementary Figure 14: Simulations of GWAS sample size by number of discovered variants, AUROC and variance explained using the PACG European meta-analysis

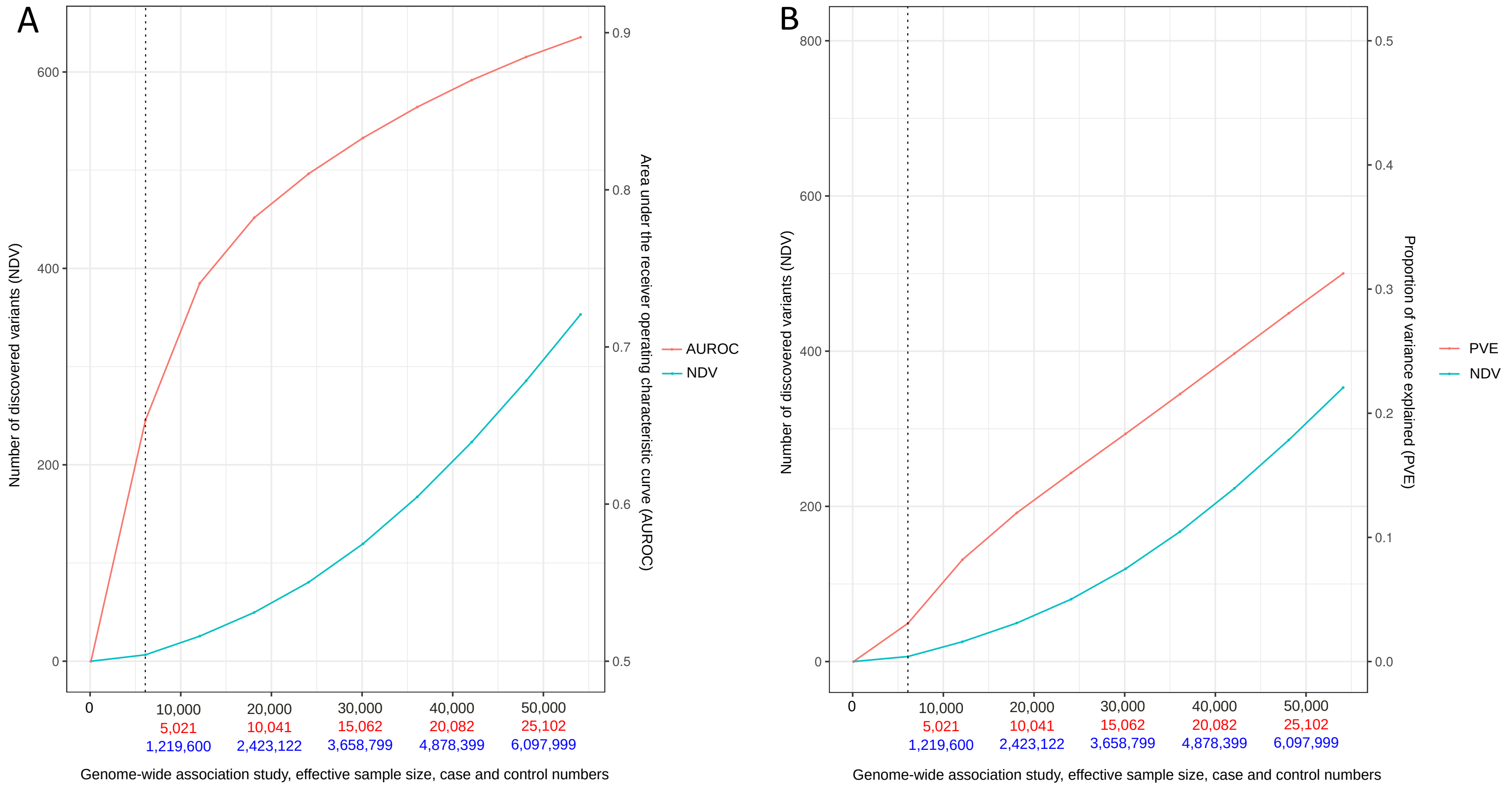

Simulations are based on the PACG European meta-analysis genome-wide association study (GWAS). Plots A and B show the simulated number of discovered variants (NDV) associated with PACG in blue (left-hand Y axes) by various measures of sample size (X axes). Plot A shows the area under the receiver operating characteristic (AUROC) curve in red (right-hand Y axis). Plot B shows the proportion of genetic variance explained (PVE) in red (right-hand Y axis). Figures on the X axes are: the GWAS effective sample size (ESS, top line in black); estimated number of PACG cases (middle line in red); estimated number of controls (bottom line in blue), calculated using the proportion of PACG cases and controls in the PACG European meta-analysis shown as a dashed line on the plots (ESS = 6,340, cases = 3,183 cases, controls = 773,214). Simulations were generated using GENESIS [6].

Supplementary Figure 15: Principal Component Analysis of replication cohorts for the determination of European ancestry

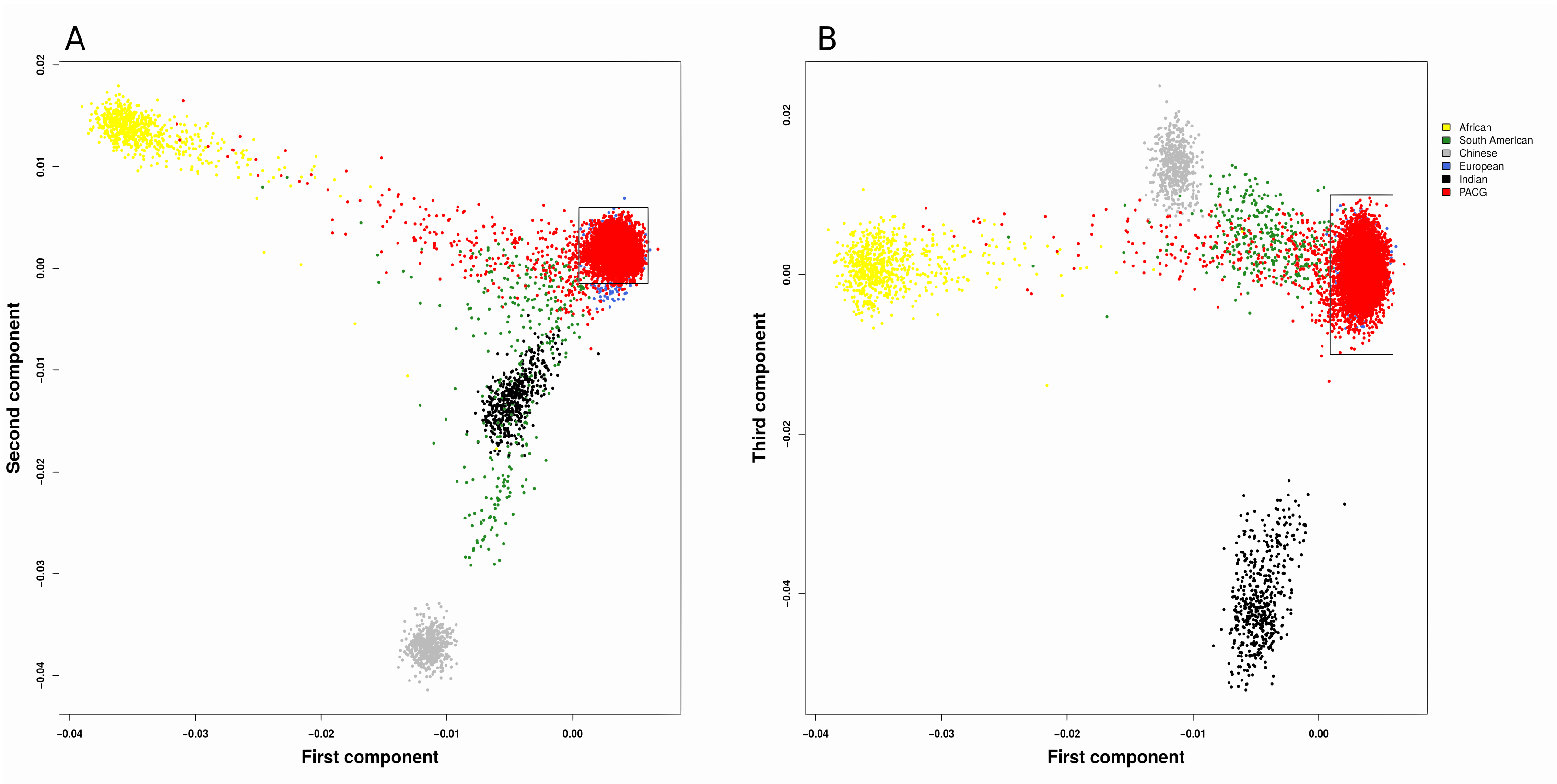

Principal components (PCs) analysis of PACG cases and controls from study replication cohorts Italy, USA, UK, Australia and Brazil (red points) overlaid with multi-ancestry 1000 Genomes Project data. **A** First and second PCs. **B** First the third PCs. Rectangular area was used to identify participants outside the European cluster who were excluded from subsequent analyses.

Supplementary Figure 16: Quantile-quantile plot and lambda statistics for discovery GWAS and European meta-analysis

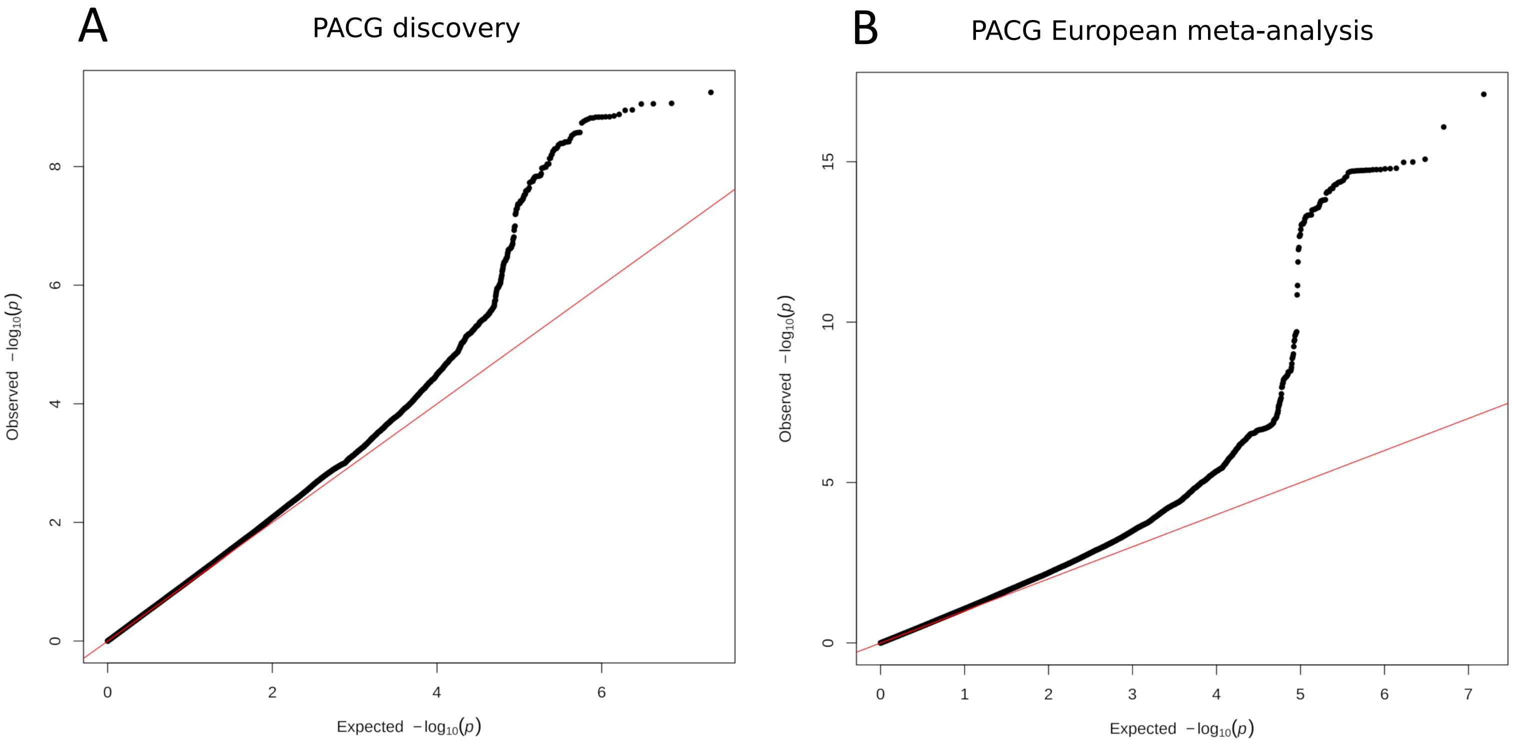

Total Observed scale h2: 0.0047 (0.0013)
Lambda GC: 1.0557
Mean χ2: 1.0529
Intercept: 1.0152 (0.0081)
Ratio: 0.2878 (0.1527)

Total Observed scale h2: 0.0111 (0.0012)
Lambda GC: 1.0988
Mean χ2: 1.1132
Intercept: 1.0138 (0.0076)
Ratio: 0.1221 (0.0668)

A Quantile-quantile (QQ) plot for the UK Biobank discovery cohort. **B** QQ plot for the European meta analysis of seven European populations. GWAS, genome-wide association study.

Supplementary Figure 17: Manhattan plot for GWAS of the UK Biobank discovery

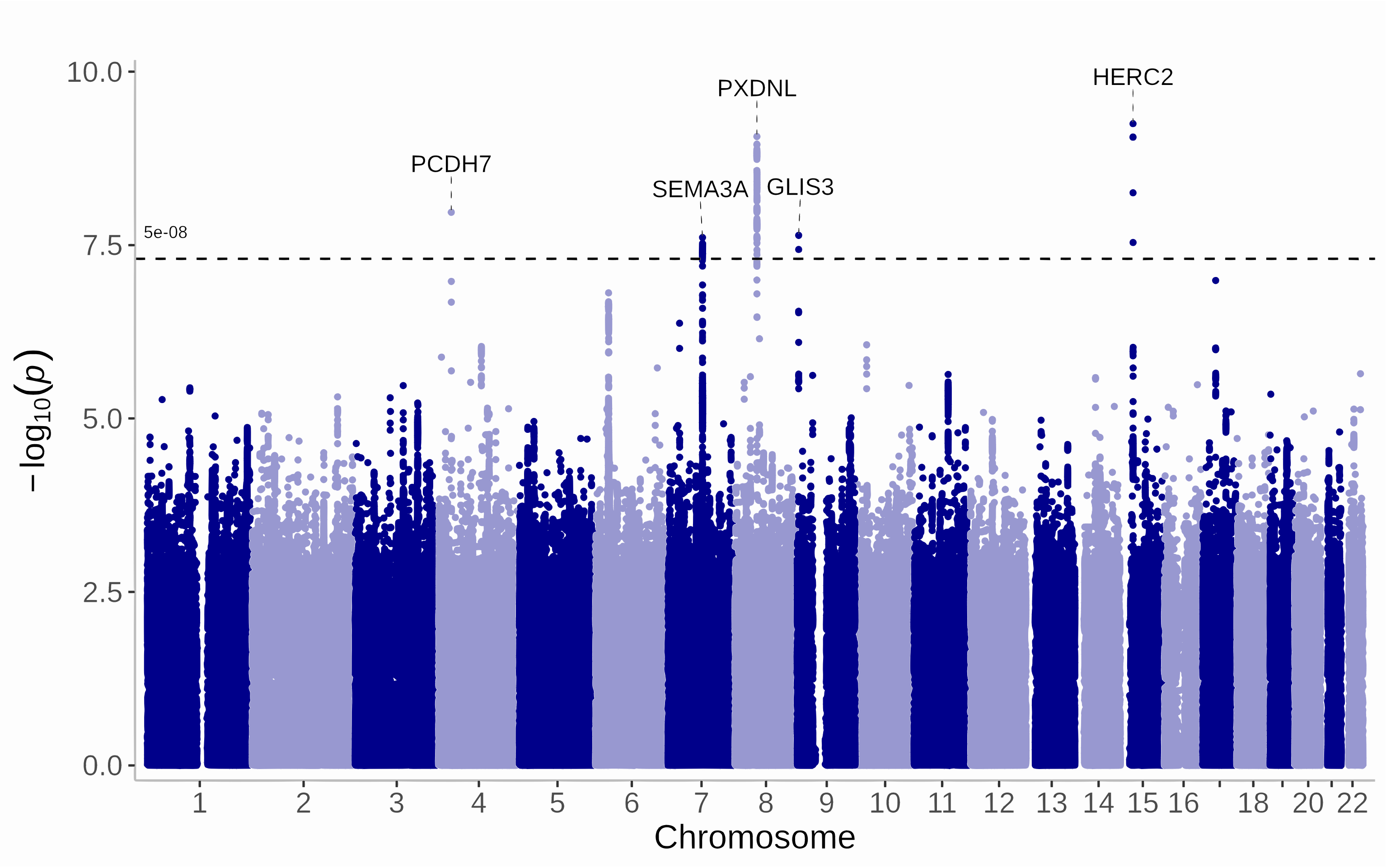

Manhattan plot using summary statistics from the UK Biobank discovery cohort of 1,564 PACG cases and 439,185 controls. GWAS associations used logistic regression with significance level $P < 5 \times 10^{-8}$ to account for multiple comparisons, and the Wald test statistic with two-sided testing. GWAS, genome-wide association study.

Supplementary note

1. Inclusion Criteria and PACG definitions for replication cohorts from Italy, UK, USA, Australia and Brazil [5]

1. Patients with acute primary angle-closure (APAC) or primary angle-closure glaucoma (PACG) were recruited
2. Informed consent
3. Age more than 21 years

1.1 Definition for Acute Primary angle-closure (APAC)

Previous APAC was defined as the presence of at least two of the following symptoms: ocular or periocular pain, nausea or vomiting or both, and an antecedent history of intermittent blurring of vision; a presenting IOP of more than 28 mmHg on Goldmann applanation tonometry; and the presence of at least three of the following signs: conjunctival injection, corneal oepithelial edema, mid-dilated non-reactive pupil, and shallow anterior chamber.

1.2 Definition for Primary angle-closure glaucoma (PACG)

Defined for cases of gonioscopic angle-closure (at least 180 degrees of angle-closure in which the trabecular meshwork was not visible on gonioscopy) with glaucomatous optic neuropathy. This is defined as disc excavation with loss of neuroretinal rim tissue and a vertical cup: disc ratio greater than the 97.5 percentile of the population (0.7), when examined with a 78D bio-microscopic lens. In addition, for PACG diagnosis, there was the presence of visual field loss detected with static automated white-on-white threshold perimetry (program 24-2 SITA, model 750, Humphrey Instruments, Dublin, Ca) that is consistent with glaucomatous optic nerve damage. This is defined as Glaucoma Hemifield test outside normal limits and/or an abnormal pattern standard deviation with $P<5\%$ occurring in the normal population.

1.3 Cohort and specific recruitment details

Italy: PACG cases were enrolled at the Dipartimento di Scienze Chirurgiche – Università di Torino, Torino, Italy. All patients and controls were unrelated Italians, all whose known ancestors were of Italian origin. The Comitato Etico Interaziendale A.O.U. San Giovanni Battista di Torino approved the study.

USA: Patients with PACG were enrolled from the New York Eye and Ear Infirmary, New York University. The controls were enrolled from the New York Eye and Ear Infirmary as well as University of Iowa. The study was approved by the IRB of the New York Eye and Ear Infirmary of Mount Sinai, New York, NY. All participants from Iowa provided informed consent and the study were approved by the University of Iowa’s IRB Board.

Australia: Participants were recruited from ophthalmology clinics in Australia. Ethical approval was obtained from the human research ethics committees of the Southern

Adelaide Health Service/Flinders University, and the study was conducted in accordance with the Declaration of Helsinki and its subsequent revisions. Informed written consent was obtained from each individual. The Australian cohort is of self-reported Caucasian ethnicity. The Australian control cohort was obtained from the Blue Mountain Eye Study, as previously described.

Brazil: They were recruited at the University of Campinas Clinical Hospital. Ethical approval was granted by the University of Campinas Research Ethics Committee, Campinas, Sao Paolo, Brazil as Certificate of Presentation for Ethical Appreciation (Certificado de Apresentação para Apreciação Ética or CAAE): 76347317.0.0000.5404 to Dr. Mônica B Melo.

United Kingdom: All subjects were of UK European descent and were recruited from Moorfields Eye Hospital, London; the Oxford Eye Hospital, John Radcliffe Hospital, Oxford University Hospitals NHS Foundation Trust (sample collection sponsored by University College London); the Department of Ophthalmology, University of Nottingham, as well as the BMI Park Hospital Nottingham. This study was approved by the Nottingham Research and Ethics Committee and the East Central London Research and Ethics Committee. The controls comprised 4703 healthy individuals of UK European descent, recruited and genotyped by the Wellcome Trust Case-Control Consortium 2.

2. Inclusion Criteria and PACG definitions for FinnGen and the EPIC-Norfolk Eye Study

FinnGen: Disease outcomes were created by combining data coded using ICD and the Anatomical Chemical Therapeutic (ACT) from one or more nationwide health registers in Finland[42]. PACG was defined using Hospital discharge and death certificates from codes ICD-10 H40.2, ICD-9 3652 and ICD-8 37500|37511 Finregistry. Nationwide electronic health registers hold historical information on hospitalisations, prescription drug purchases (not including hospital administered medications), medical procedures or deaths. Linking used the unique personal identity code (PIC) which is assigned to every permanent resident of Finland. Nine regional biobanks, the Finnish Institute for Health and Welfare (THL), the Blood Service and the Terveystalo biobanks provide samples to FinnGen. GWAS summary statistics can be obtaining by completing a form as described in this link: https://www.finngen.fi/en/access_results

EPIC-Norfolk Eye Study: Participants were recruited from 35 general practices in Norfolk, England and were invited at baseline to take part in this prospective cohort study. The EPIC-Norfolk Eye Study was completed at the third round of health examinations on 8623 participants in the age range 48–92 years. PACG, which included primary angle closure (PAC) cases, was ascertained by clinical examination (including gonioscopy) by a glaucoma specialist. The study was carried out following the principles of the Declaration of Helsinki and the Research Governance Framework for Health and Social Care. It was approved by the Norwich Local Research Ethics Committee (05/Q0101/191) and East Norfolk & Waveney National Health Service (NHS) Research Governance Committee (2005EC07L). All participants gave written, informed consent.

Supplementary References

1. Ghoussaini, M. *et al.* Open Targets Genetics: systematic identification of trait-associated genes using large-scale genetics and functional genomics. *Nucleic Acids Res* **49**, D1311–D1320 (2020).
2. Jiang, C. *et al.* A multiethnic genome-wide analysis of 19,420 individuals identifies novel loci associated with axial length and shared genetic influences with refractive error and myopia. *Frontiers in Genetics* **14**, (2023).
3. Simcoe, M. *et al.* Genome-wide association study in almost 195,000 individuals identifies 50 previously unidentified genetic loci for eye color. *Science Advances* **7**, eabd1239 (2021).
4. Hysi, P. G. *et al.* Meta-analysis of 542,934 subjects of European ancestry identifies new genes and mechanisms predisposing to refractive error and myopia. *Nat Genet* **52**, 401–407 (2020).
5. Khor, C. C. *et al.* Genome-wide association study identifies five new susceptibility loci for primary angle closure glaucoma. *Nat Genet* **48**, 556–562 (2016).
6. Gogarten, S. M. *et al.* Genetic association testing using the GENESIS R/Bioconductor package. *Bioinformatics* **35**, 5346–5348 (2019).
