# Supplementary material for: GWAS for primary angle-closure glaucoma identifies loci related to ocular biometry and morphology
Source: Nat Commun. 2025 Nov 14;16:10003. doi: 10.1038/s41467-025-64949-z (PMC12618631; doi:10.1038/s41467-025-64949-z)
Supplement: Supplementary file 2 — Description of Additional Supplementary Files [file 41467_2025_64949_MOESM2_ESM.pdf]

Description of Additional Supplementary Files

File name: Supplementary data 1 PACG_GWAS_Supplementary_DATA1_20250731.xlsx

Description: Lead variants from the Multi-Trait Analysis for GWAS analysis

Results are presented for the genome-wide significant loci associated with PACG in the Multi-Trait Analysis for GWAS (MTAG) analysis using summary statistics for PACG (current European ancestry meta-analysis) and refractive error (Hysi et al. 2020 [4]). All lead variants were genome-wide significant ($P < 5 \times 10^{-8}$) in the MTAG analysis and are highlighted in bold if they were also genome-wide significant in our European ancestry meta-analysis. Genome positions refer to GRCh37. GWAS, genome-wide association study; PACG, primary angle-closure glaucoma; SE, standard error; SNP, single nucleotide polymorphism. Single ticks and double ticks against ocular biometry or refraction, iris colour or morphology, or glaucoma represent $5 \times 10^{-8} \leq P < 5 \times 10^{-4}$ and $P < 5 \times 10^{-8}$ respectively for each variant having an association with a related trait under each heading, as reported by Open Targets Genetics [1].
