# Supplementary material for: GWAS for primary angle-closure glaucoma identifies loci related to ocular biometry and morphology
Source: Nat Commun. 2025 Nov 14;16:10003. doi: 10.1038/s41467-025-64949-z (PMC12618631; doi:10.1038/s41467-025-64949-z)
Supplement: Supplementary file 4 — Reporting Summary [file 41467_2025_64949_MOESM4_ESM.pdf]

nature portfolio

Corresponding author(s): Robert Luben

Last updated by author(s): Sep 2, 2025

Reporting Summary

Nature Portfolio wishes to improve the reproducibility of the work that we publish. This form provides structure for consistency and transparency in reporting. For further information on Nature Portfolio policies, see our Editorial Policies and the Editorial Policy Checklist.

Statistics

For all statistical analyses, confirm that the following items are present in the figure legend, table legend, main text, or Methods section.

n/a	Confirmed	
☐	☒	The exact sample size (*n*) for each experimental group/condition, given as a discrete number and unit of measurement
☒	☐	A statement on whether measurements were taken from distinct samples or whether the same sample was measured repeatedly
☐	☒	The statistical test(s) used AND whether they are one- or two-sided<br>*Only common tests should be described solely by name; describe more complex techniques in the Methods section.*
☐	☒	A description of all covariates tested
☐	☒	A description of any assumptions or corrections, such as tests of normality and adjustment for multiple comparisons
☐	☒	A full description of the statistical parameters including central tendency (e.g. means) or other basic estimates (e.g. regression coefficient) AND variation (e.g. standard deviation) or associated estimates of uncertainty (e.g. confidence intervals)
☐	☒	For null hypothesis testing, the test statistic (e.g. F, t, r) with confidence intervals, effect sizes, degrees of freedom and P value noted<br>*Give P values as exact values whenever suitable.*
☒	☐	For Bayesian analysis, information on the choice of priors and Markov chain Monte Carlo settings
☒	☐	For hierarchical and complex designs, identification of the appropriate level for tests and full reporting of outcomes
☐	☒	Estimates of effect sizes (e.g. Cohen's *d*, Pearson's *r*), indicating how they were calculated

Our web collection on statistics for biologists contains articles on many of the points above.

Software and code

Policy information about availability of computer code

Data collection	No software was used
Data analysis	No custom software was used.

For manuscripts utilizing custom algorithms or software that are central to the research but not yet described in published literature, software must be made available to editors and reviewers. We strongly encourage code deposition in a community repository (e.g. GitHub). See the Nature Portfolio guidelines for submitting code & software for further information.

Data

Policy information about availability of data

All manuscripts must include a data availability statement. This statement should provide the following information, where applicable:

- Accession codes, unique identifiers, or web links for publicly available datasets
- A description of any restrictions on data availability
- For clinical datasets or third party data, please ensure that the statement adheres to our policy

We obtained data files containing directly called and imputed genetic data from UK Biobank and EPIC-Norfolk. Cohorts from Australia, USA, UK, Italy and Brazil provided directly called genetic data. A UK Biobank dataset containing hospital ICD10 codes was used to derive PACG and contained age and sex. A dataset from the EPIC-Norfolk Eye Study contained PACG outcome, age and sex. A dataset of GWAS summary statistics for PACG was obtained from FinnGen.

Access to UK Biobank data requires an application in order to protect the privacy of participants and to conform to confidentiality and data governance policies. Requests for access to UK Biobank data should be made to the UK Biobank Access Management Team (access@ukbiobank.ac.uk). Access to EPIC-Norfolk data requires an application in order to protect the privacy of participants and to conform to confidentiality and data governance policies. Requests for access to EPIC-Norfolk data can be made via https://www.epic-norfolk.org.uk/for-researchers/data-sharing/data-requests. Access to FinnGen summary statistics can be made via https://www.finngen.fi/en/access_results. Contact details of national replication cohorts can be found in the Supplementary Note. The summary-level data generated in this study are provided in the Source Data file.

Research involving human participants, their data, or biological material

Policy information about studies with human participants or human data. See also policy information about sex, gender (identity/presentation), and sexual orientation and race, ethnicity and racism.

Reporting on sex and gender	Men and women were combined for all analyses as indicated in our study design. Genetic analyses (GWAS) and linear modeling of phenotypes were adjusted for sex. Biological sex was used where available and derived from genetic data if necessary. Individuals where biological sex differed from self reported sex or sex held in health service databases were excluded.
Reporting on race, ethnicity, or other socially relevant groupings	Cohorts used in the study were identified either by their name (UK Biobank, FinnGen and EPIC-Norfolk) or by the name of the country where the cohort originated. Participants with European ancestry were identified in the discovery and replication cohorts using principal components (PCs) analysis, where the selection of these cohort was based on the likelihood of their having substantial numbers of people with European ancestry. Cohorts from countries with substantial Asian ancestry populations were grouped as participants of Asian ancestry. Genetic models were adjusted for the first 10 PCs to control for potential confounding by ancestry, while also being adjusted for age and sex.
Population characteristics	The UK Biobank discovery cohort comprised of 202,164 men and 238,585 women with mean age 56.8 years and age range 39 to 73 years. There are 1564 PACG cases and 439185 controls. The combined replication cohorts (excluding FinnGen, for which only summary statistics were available) has 3962 men and 4571 women. There are 1619 replication cases and 334031 controls. Further details of the population characteristics are shown in Figure 1, Study Design and the Methods section.
Recruitment	Methods of recruitment varied by cohort, with some using community-based populations and others through hospitals. UK Biobank participants were recruited from 22 centres throughout the UK. FinnGen participants were recruited from the Finnish National Institute for Health and Welfare and from hospital biobanks in Finland. EPIC-Norfolk participants were recruited from 35 participating general practices in Norfolk. Details of the other cohorts are shown in the Supplementary Note.
Ethics oversight	UK Biobank received ethics approval from the North West Multi-center Research Ethics Committee. Details of ethical approvals of other studies are shown in the Supplementary Note.

Note that full information on the approval of the study protocol must also be provided in the manuscript.

Field-specific reporting

Please select the one below that is the best fit for your research. If you are not sure, read the appropriate sections before making your selection.

☒ Life sciences ☐ Behavioural & social sciences ☐ Ecological, evolutionary & environmental sciences

For a reference copy of the document with all sections, see nature.com/documents/nr-reporting-summary-flat.pdf

Life sciences study design

All studies must disclose on these points even when the disclosure is negative.

Sample size	In the UK Biobank discovery GWAS, 1564 primary angle-closure glaucoma (PACG) cases and 439,185 controls used. In the European meta-analysis 3183 PACG and 773,214 controls were used. In the multi-ancestry meta-analysis 9217 PACG cases and 788,285 controls were used. UK Biobank was used for discovery GWAS since it is the largest available European cohort with PACG derived from medical coding. The number of replication participants was maximised by using all available independent national cohorts with populations having mainly European ancestry.
Data exclusions	Exclusions in European ancestry discovery GWAS were made of participants who did not have European genetic ancestry. Genetic quality control excluded individuals if their missing call rates exceeded 0.1. Variants with Hardy-Weinberg equilibrium exact test having a P-value ≤10^-15, a lower minor allele count bound ≤ 100, a minor allele frequency (MAF) ≤ 0.01 or an information quality score ≤ 0.8 were excluded. Genotypes were excluded from the meta-analyses where imputation information scores were < 0.4 and MAF < 0.05. EPIC-Norfolk study controls were excluded if they were known to have other forms of glaucoma or were glaucoma suspects.
Replication	Associations using all available data were presented with meta-analyses using to maximize discovery power. Although high thresholds for significance were used and multiple levels of replication were performed, further replication in subsequent studies should be performed to confirm the findings. Replication included comparison of the discovery cohort with 6 independent replication cohorts and an assessment of prediction accuracy in another independent cohort.
Randomization	Randomization was not applicable since the study design was observational.
Blinding	Blinding was not needed since the study design was observational and not experimental.

Reporting for specific materials, systems and methods

We require information from authors about some types of materials, experimental systems and methods used in many studies. Here, indicate whether each material, system or method listed is relevant to your study. If you are not sure if a list item applies to your research, read the appropriate section before selecting a response.

Materials & experimental systems

n/a	Involved in the study
☒	☐ Antibodies
☒	☐ Eukaryotic cell lines
☒	☐ Palaeontology and archaeology
☒	☐ Animals and other organisms
☒	☐ Clinical data
☒	☐ Dual use research of concern
☒	☐ Plants

Methods

n/a	Involved in the study
☒	☐ ChIP-seq
☒	☐ Flow cytometry
☒	☐ MRI-based neuroimaging

Plants

Seed stocks	*Report on the source of all seed stocks or other plant material used. If applicable, state the seed stock centre and catalogue number. If plant specimens were collected from the field, describe the collection location, date and sampling procedures.*
Novel plant genotypes	*Describe the methods by which all novel plant genotypes were produced. This includes those generated by transgenic approaches, gene editing, chemical/radiation-based mutagenesis and hybridization. For transgenic lines, describe the transformation method, the number of independent lines analyzed and the generation upon which experiments were performed. For gene-edited lines, describe the editor used, the endogenous sequence targeted for editing, the targeting guide RNA sequence (if applicable) and how the editor was applied.*
Authentication	*Describe any authentication procedures for each seed stock used or novel genotype generated. Describe any experiments used to assess the effect of a mutation and, where applicable, how potential secondary effects (e.g. second site T-DNA insertions, mosiacism, off-target gene editing) were examined.*
